# Heavy Metals in Notifications of Rapid Alert System for Food and Feed

**DOI:** 10.3390/ijerph15020365

**Published:** 2018-02-20

**Authors:** Marcin Pigłowski

**Affiliations:** Department of Commodity and Quality Management, Faculty of Entrepreneurship and Quality Science, Gdynia Maritime University, Morska 81-87, 81-225 Gdynia, Poland; ktizj@wpit.am.gdynia.pl or m.piglowski@wpit.am.gdynia.pl; Tel.: +48-58-5586-295

**Keywords:** food safety, heavy metals, RASFF

## Abstract

Heavy metals represent the fourth most often notified hazard category in the Rapid Alert System for Food and Feed (RASFF) from 1980–2016. The goal of the study was to examine the similarities in notifications of particular heavy metals within the RASFF year, product category, notifying country, country of origin, notification basis, notification type, distribution status, risk decision, and action taken, taking into account the particular product type, such as food, food contact material, and feed. The data originated from the RASFF database. Cluster analysis on pivot tables was applied using joining and two-way joining methods. Most notifications concerned food, in which the highest number were related to mercury, cadmium, chromium, lead, arsenic, and nickel. Notifications were mainly related to fish and food contact materials, in addition to fruits and vegetables, seafood, and dietetic food. The number of notifications decreased in 2015 and 2016. The majority of products were notified by Italy, Spain, Germany, and France. The notified products originated mainly from China and Spain. The notification was usually based on official controls on the market, as well as border controls. The notification types were mainly information, alert, and border rejections. Products were not frequently distributed due to distribution restriction to the notifying country or the possibility of distribution to the market. A risk decision was not usually made. The taken actions included re-dispatch of products, withdrawal from the market, or destruction. The data on heavy metals from the RASFF database can help European and national authorities in shaping public health.

## 1. Introduction

Morgan [1] noted that heavy metal contamination of foods may occur at various stages of production, from growth to consumption. Sources and toxicity of heavy metals and mechanisms of their uptake by microorganisms and bioremediation have been widely discussed [2,3,4,5,6,7,8,9,10,11]. However, heavy metals are indestructible, and therefore can concentrate in the food chain and eventually accumulate in the human body [2,12]. Filipiak-Florkiewicz et al. [13] mentioned that neuroendocrine disruptions caused by heavy metals and other organic pollutants can be associated with their neurotoxicity and obesity prevalence.

In contaminated soil and water, the following elements can occur: arsenic, cadmium, chromium, mercury, lead, and zinc [12]. Tóth et al. stated that in the European Union (EU) countries, concentrations of heavy metals in soils are relatively—but not alarmingly—high [14]; however, they suggested that Western European soils should be assessed and monitored in detail [15]. Bao et al. [16] reported that the concentrations of mercury, lead, and copper should be monitored in areas that are engaged in treated sewage irrigation, in order to prevent these heavy metals from entering the food chain and presenting a health risk. Similarly, Hassan et al. [17] mentioned that regular monitoring of heavy metals from industrial and domestic effluents, irrigation waters, and crops is essential to preventing their excessive accumulation in the food chain. Wang et al. [18] also drew attention to the necessity of regular monitoring of heavy metal contamination in crops grown in wastewater-irrigated sites, adding that wastewater technology should be perfected to improve the removal of heavy metals. In turn, Islam et al. [19] stated that agronomic practices such as fertilizers, water management, and crop rotation can affect the bioavailability and crop accumulation of heavy metals. Solanki and Dhankhar [20] suggested that understanding the mechanisms by which plants defend themselves against heavy metal stress could be helpful in the generation and selection of heavy metal-tolerant plants, which could be grown in fields containing high levels of heavy metals and intended for human consumption.

However, the prevalence of heavy metals in the aquatic environment is noticed particularly often. Fish generally accumulate heavy metal contaminants [21,22]. Jakimska et al. [23] noted that the harmfulness of heavy metals to marine organisms—resulting from bioaccumulation in tissues and organs—relates not only to the extent of metals’ absorption/excretion, but also to their biochemical role in metabolic processes. Olgunoğlu [24] stressed that heavy metal pollution in the aquatic environment can be harmful to human health; therefore, it is necessary to understand and control their hazard levels in seafood. In turn, Mania et al. [25] paid particular attention to the necessity of selecting raw materials by manufacturers of infant foods, including fish-based products, in addition to food containing rice, vegetables, or meat. Islam et al. [26] determined the contamination of heavy metals in both fish and vegetables.

Council Regulation No. 315/93 [27] established procedures for determining the levels of contaminants in food. The maximum levels for certain heavy metals in foodstuffs were set in Commission Regulations No. 1881/2006 [28] and No. 629/2008 [29]. Regulation No. 1935/2004 [30] referred to metals in materials and articles intended to come in contact with food. In turn, the maximum content of heavy metals in feed was determined in directive 2002/32 [31]. However, heavy metals are still one of the most often notified hazards in the Rapid Alert System for Food and Feed (RASFF). The number of notifications of heavy metals in the RASFF was surpassed only by notifications of mycotoxins, pathogenic micro-organisms, and pesticide residues [32,33,34,35].

Currently, the legal basis for RASFF is Regulation No. 178/2002, which was created in 1979 to provide general principles and requirements of food law, establishing the European Food Safety Authority, and laying down procedures in matters of food safety. The RASFF enables the exchange of information on hazards in food between its members, which include the EU countries, the European Commission, the European Food Safety Authority, the European Free Trade Association Surveillance Authority, Norway, Liechtenstein, Iceland, and Switzerland. There are four notification types: alerts, information (sent in 1991–2011) or information for attention and information for follow-up (sent from 2011), border rejections, and news. Alert notifications are sent when food or feed present a serious risk on the market and rapid action (e.g., market withdrawal) is necessary. First, the RASFF member transmits information through the system to other members to confirm whether the product is on their market, so that they can also take relevant actions. Information notifications are sent when food or feed presenting risk is on the market but has not reached other RASFF members’ market, is no longer present on their market (information for attention), or risk does not require rapid actions (information for follow-up). Border rejections relate to food or feed consignments that have been tested in terms of risk and rejected at the external border of the EU, as well as the wider European Economic Area (EEA). Notifications are sent to all EEA border posts to introduce controls and to ensure that the rejected product does not re-enter the EEA through another border post. News notifications are transmitted when information related to food or feed safety can be interesting for control authorities but are not communicated as an alert or information notification [32].

The data on heavy metals given in annual RASFF reports are of a general and casual nature or are only short case studies. Therefore, the goal of this study was to examine the similarities in notifications of particular heavy metals in the RASFF within year, product category, notifying country, origin country, notification basis, notification type, distribution status, risk decision, and action taken, taking into account particular product type (i.e., food, food contact material, and feed).

## 2. Materials and Methods

The data related to notifications on heavy metals originated from the RASFF database and were composed of 3436 notifications from 1980 to 2016. They were collected in three product types: food (for human consumption), food contact material (e.g., containers, packaging, kitchenware), and animal feed. The data concerned: heavy metal (name), year, product category, notifying country (in the database: notified by), origin country (in the database: origin within countries concerned), notification basis, notification type, distribution status, risk decision, and action taken.

The vast majority of notifications reported one heavy metal (86%). Therefore, in the present study, the first heavy metal among all the metals listed in the notification was adopted (the co-occurrence of two heavy metals examined only within product categories). A similar approach was used in the case of the origin country, i.e., only the first country from the mentioned origin countries was adopted, directly preceded by the expression “from”. So, the countries from where raw materials originated, in which the product was manufactured, processed, or packaged (in rare cases, if it was separately indicated), countries—through which the product was transported or other countries in which the product was distributed—were omitted. The data concerned also product categories, which have been already obsolete (names of these categories have been changed or they were included in another category during the RASFF development). In some cases, data on heavy metal, notification basis, distribution status, action taken were not given; therefore, they were completed by the expression “not specified”.

Pivot tables and a cluster analysis were used to conduct a detailed examination of the similarities in the number of notifications of particular heavy metals, year, product category, notifying country, origin country, notification basis, notification type, distribution status, risk decision, and action taken within food, food contact materials, and feed. Heavy metals were adopted as variables, but the year, product category, notifying country, origin country, notification basis, notification type, distribution status, risk decision, and action taken were adopted as cases. The cluster analysis included joining (tree clustering) with the following settings: linkage rule—Ward’s method (vertical icicle plots) and distance measure—Euclidean distance and two-way joining (block clustering) using Statistica 12 (12.5.192.0, StatSoft, Tulsa, OK, USA). The figures resulting from the cluster analysis were created on the basis of pivot tables and placed in Supplementary Materials in pairs. On the left side of each figure, part (a) presents joining, and on the right side, part (b) presents two-way joining. Part (a) is complemented and explained in detail in part (b) to uncover meaningful patterns of clusters. The darker (brown or red) blocks in the two-way joining indicate notifications which occurred in the greatest number. However, the character of these figures was auxiliary, and showed the most important similarities.

## 3. Results and Discussion

Without dividing into particular product types, the highest number of notifications were related to mercury (36%), cadmium (27%), chromium (14%), lead (9%), arsenic (6%), nickel (4%), manganese, tin, zinc (each of them about 1%), and iron, cobalt, antimony, copper, selenium, and elements not specified (below 1%), respectively. The highest number of notifications occurred from 2011 to 2014, but this number decreased in 2015 and 2016. Occurrences of heavy metals were notified in fish and fish products (38%), food contact materials (28%), fruits and vegetables (6%), seafood (i.e., crustaceans and products thereof, 5%, cephalopods and products thereof, 4%, molluscs and products thereof, 3%), and dietetic foods, food supplements, and fortified foods (4%). Products were notified mainly by Italy (47%), Spain (10%), Germany (7%), France (6%), and also Belgium, Poland, Slovenia, the United Kingdom, Finland, Greece, the Netherlands, and the Czech Republic. The notified products originated from China (25%) and other Far East Asia countries (i.e., India, 4%, Vietnam, 4%, Thailand, 3%, Indonesia, 3%), and from Spain (13%) and France (3%). The notification basis was mainly official control on the market (41%), which caused alert or information notifications and border control when the consignment was detained, which was linked with border rejections, but consignments could also be released (9%). In 4% of cases, the notification basis was the company’s own check; however, there was no specification in 6% of the cases. The notification type was information (34%), information for attention (13%), information for follow-up (3%), alert (27%), and border rejections (24%). The distribution status was indicated as “no distribution” (25%), “distribution restricted to the notifying country” (16%), and “distribution on the market (possible)” (14%). The risk decision was most often not made (71%); only in 21% was it specified as serious, and in 8% of cases as not serious. The notified products were re-dispatched (22%), withdrawn from the market (16%), or destroyed (8%); however, other actions were also taken (e.g., official detention, import not authorized, seizure, and recall from consumers).

However, Table 1, Table 2 and Table 3—created on the basis of the results obtained from the pivot tables—present in detail the number of notifications of heavy metals taking into consideration the year, product category, notifying country, origin country, notification basis, notification type, distribution status, risk decision, and action taken, and the particular product type (e.g., food, food contact material, and animal feed). The numbers above the mean, starting with highest number, are given in brackets. Some product categories were obsolete (molluscs and products thereof, wild caught fish and products thereof, animal nutrition, feed for food-producing animals, farmed crustaceans, and products thereof). In some cases, the distribution status and action taken were not specified. In other cases, there were many values with similar numbers, which caused difficulties in interpretation. This particularly applied to origin country and year, distribution status, and action taken. In such cases, all these values were listed in the tables mentioned above, but in the discussion below only the value(s) with the highest number(s) were indicated. The preliminary results of the similarities in notifications of heavy metals are presented in Figures S1–S26 in part (a), where the cluster analysis using joining was applied, and in Figures S1–S26 in part (b), where two-way joining was used. These results are presented within particular product type (food, food contact material, and feed) and the concerned year, product category, notifying country, origin country, notification basis, notification type, distribution status, risk decision, and action taken.

The vast majority were notifications on food (2509, 73% of all notifications). Above the mean value within this product type, there were notifications of mercury (1203), cadmium (741), and lead (215) (Table 1). However, within food there were also notifications on arsenic, chromium, tin, nickel, zinc, iron, manganese, antimony, copper, and not specified elements.

Notifications related to heavy metals in food were similar for mercury and chromium, creating a cluster in the case of year (Figure S1a), notifying country (Figure S3a), notification basis (Figure S5a), notification type (Figure S6a), distribution status (Figure S7a), risk decision (Figure S8a), and action taken (Figure S9a). The second cluster consisted of two sub-clusters: chromium, lead, and arsenic, and other heavy metals. However, for product category (Figure S2a) and origin country (Figure S4a), mercury created a one-element cluster, which indicated that notifications of this heavy metal were a particular focus here.

The number of notifications on mercury maintained on a high level (above 100 per year), but notifications on cadmium and lead decreased in recent years (Figure S1b). Notifications on food related mainly to mercury and cadmium in fish and fish products were shown in Figure S2b. Cadmium was also notified in seafood, i.e., cephalopods and products thereof, crustaceans and products thereof, and molluscs and products thereof. In turn, lead was notified in fruits and vegetables, dietetic foods, food supplements, fortified foods, and food contact materials. These products were notified by Western European countries, mainly by Italy (Figure S3b). In the case of mercury, the origin country was mainly Spain (products were notified on the basis of official controls on the market), but also Asian countries, as in the case of cadmium and lead (where the notification basis was mainly border control) (Figures S4b and S5b). Notifications were notified as alert (mercury and lead) or information (mercury, cadmium and lead), as shown in Figure S6b. Distribution was indicated as restricted to the notifying country, on the market (possible); however, distribution status was also referred to as “no distribution” or was not specified (Figure S7b). The decision on risk was usually not made (Figure S8b), and the products were mainly withdrawn from the market, re-dispatched, or destroyed (Figure S9b).

In the case of food contact material (776, 23% of all notifications), above the mean value there were notifications on chromium (387), cadmium (139), and nickel (124) (Table 2). In this product type there were also notifications on lead, manganese, cobalt, iron, zinc, and arsenic.

Notifications on heavy metals in food contact material were focused mainly on chromium, which created a one-element cluster (part (a) in Figures S10–S17). The second cluster consisted of sub-clusters, where notifications had a similar character for both cadmium and nickel and for lead and manganese (year—Figure S10a, origin country—Figure S12a, notification basis—Figure S13a, distribution status—Figure S15a, and action taken—Figure S17a).

The number of notifications on chromium and nickel decreased and those on cadmium increased in recent years (Figure S10b). The notifications centered only on food contact materials, and therefore generating the figure for product category within food contact material using two-way joining was not possible. These products were notified by Italy in the case of chromium and nickel, and by Poland and Germany in the case of cadmium (Figure S11b), and were from China (Figure S12b). The notification origin in chromium and nickel was border control, and the notification action was border rejection. In the case of cadmium, the notification basis was official control on the market, and the notification type was alert or information for attention (Figures S13b and S14b). The products containing chromium and nickel were not distributed or not placed on the market. In the case of products containing cadmium, distribution was restricted to the notifying country; however, products could be also distributed to other countries (Figure S15b). The decision on risk was mainly indicated as “undecided”, but risk could also be “serious”—in the case of products with cadmium—or “not serious”—products with nickel—Figure S16b. The products with chromium and nickel were mainly re-dispatched, and products with cadmium were withdrawn from the market (Figure S17b).

The smallest number of notifications was on feed (151, 4% of all notifications), and those above the mean value were notifications for arsenic (47), cadmium (34), mercury (30), and lead (29) (Table 3). There were also notifications on zinc, iron, cobalt, and selenium.

In the case of notifications of heavy metals in feed, two clusters could usually be observed. The first covered cobalt, iron, selenium, and zinc, and the second cluster consisted of arsenic, cadmium, lead, and mercury. The similar character had notifications on cadmium and lead (year—Figure S18a, product category—Figure S19a, notifying country—Figure S20a, origin country—Figure S21a, notification basis—Figure S22a, notification type—Figure S23a, distribution status—Figure S24a, risk decision—Figure S25a, and action taken—Figure S26a). The similarities in notifications also occurred in the case of arsenic and mercury (product category—Figure S19a, notifying country—Figure S20a, origin country—Figure S21a, notification basis—Figure S22a, notification type—Figure S23a, distribution status—Figure S24a, and action taken—Figure S26a).

The number of notifications on arsenic and cadmium decreased, while mercury and lead increased in recent years (Figure S18b). In the case of each heavy metal (i.e., arsenic, cadmium, mercury, and lead), notifications focused mainly on feed materials, pet food (in the case of arsenic and mercury), and feed additives (in the case of cadmium and lead) (Figure S19b). Products with arsenic and mercury were notified mainly by Italy; however, Germany and Belgium were also active in notifying (Figure S20b). Thailand was the origin country in the case of products with arsenic and mercury, products with cadmium originated from China and Spain, and products with lead from the Netherlands and Spain (Figure S21b). The notification basis for products was mainly official control on the market, the company’s own check, or border control (Figure S22b). Information for follow-up was in the case of products with each heavy metal mentioned above (arsenic, cadmium, mercury, and lead), alert notifications occurred mainly in the case of products with arsenic and lead, border rejections in the case of arsenic and mercury, and information notification in the case of cadmium and lead (Figure S23b). Distribution status was indicated as “distribution restricted to notifying country” (products with arsenic, cadmium, mercury, and lead), “no distribution” (in the case of products with arsenic), or “distribution to other member countries” (products with arsenic, cadmium, and lead). In some cases, distribution on the market was also possible (products with cadmium and lead) or products were not placed on the market (products with mercury—Figure S24b). The risk decision was usually indicated as “undecided” (products with arsenic, cadmium, and lead) or risk was “not serious” (products with mercury—Figure S25b). Products were most frequently re-dispatched or withdrawn from the market (Figure S26b).

The second heavy metal occurred in 481 notifications (about 14% of all cases) and mainly concerned two product types—i.e., food (169 notifications) and food contact material (304 notifications). There were only eight notifications in feed. For food, there was the co-occurrence of chromium and nickel, cadmium, and lead in food contact materials (treated here as product category, not product type), cadmium and mercury in fish and fish products, arsenic and lead as well as arsenic and mercury in dietetic foods, food supplements, and fortified foods. In the case of food contact material, the co-occurrences of two heavy metals were related to cadmium and lead, chromium and nickel, and chromium and manganese.

The RASFF notifications on heavy metals in food and feed were mentioned by Cheftel [36], Ismail et al. [37], and Tlustos et al. [38]. Bouxin [39] drew attention to notifications of heavy metals in feed. However, the authors most frequently noted RASFF notifications on heavy metals in relation to fish or seafood; i.e., fish and fish products [40], fish [35], fishery products (from China) [41], swordfish and prawns [42], seafood [43], and seafood (from Vietnam) [44]. Some authors indicated a heavy metal name; i.e., mercury, cadmium, and lead in seafood [45], mercury and cadmium in seafood [46], cadmium in crabs (from France, Ireland, and the United Kingdom) [47], methylmercury in fish [48], and mercury in fish and seafood (from China) [49]. Figueora [50] noticed that RASFF notifications on cadmium in fishery products from developing countries can be a barrier to trade and can cause economic losses for these countries.

Van Boxtael et al. [51] and Uyttendaele et al. [52] mentioned RASFF notifications on heavy metals in fruits and vegetables and herbs and spices. Banach et al. [53] drew attention to notifications in herbs and spices. Van Asselt et al. [54] indicated that within the RASFF, arsenic, cadmium, lead, and mercury were notified most frequently in herbs and species. In turn, Christoper and Thompson [55] and Da Justa Neves and Caldas [56] mentioned RASFF notifications on heavy metals in dietary supplements. Baer et al. [57] drew attention to the fact that lead, cadmium, and mercury were indicated in food supplements within RASFF notifications.

Rortais et al. [42] also pointed to notifications on heavy metals related to food contact materials. Kleter et al. [46] stated that RASFF notifications on chromium and nickel related to migration from utensils from China (see also [58]). RASFF notifications on migration of lead, cadmium, and chromium from kitchen utensils were also mentioned by Elskens et al. [59] and notifications on migration of lead and nickel were noticed by Potter et al. [60].

## 4. Conclusions

Heavy metals were the fourth most frequently notified hazard category in the RASFF, after mycotoxins, pathogenic micro-organisms, and pesticide residues. The majority of RASFF notifications on heavy metals occurred in the following product types: food (73%), food contact material (23%), and feed (4%). In general, the highest number of notifications were related to mercury (36%), cadmium (27%), chromium (14%), lead (9%), arsenic (6%), and nickel (4%), respectively. Notifications focused mainly on fish and fish products (38%), food contact materials (28%), fruits and vegetables, seafood (crustaceans and products thereof, molluscs and products thereof, cephalopods and products thereof), and dietetic foods, food supplements, and fortified foods. Notifications on food related mainly to mercury, cadmium, and lead. Mercury was notified in fish and fish products, cadmium in fish and fish products or seafood, and lead in fruits and vegetables, dietetic foods, food supplements, fortified foods, and food contact materials. Notifications on food contact materials centered on chromium, cadmium, and nickel. For feed, however, the notifications were regarding arsenic, cadmium, mercury, and lead in feed materials, arsenic and mercury in pet food, and cadmium and lead in feed additives.

The number of notifications decreased from 2015 to 2016 (mercury in food was an exception, where the number of notifications maintained a high level). Products were notified mainly by Italy (47% of all notifications), Spain, Germany, and France. The highest number of notifications related to products from China (25% of all notifications) and from Spain (13%). The notifications basis was usually official controls on the market (41% of all notifications) and border controls (consignment was detained) (37%). The notification actions were information (34%), information for attention (13%), or information for follow-up (3%), alert (27%), and border rejections (24%). Products were most frequently not distributed (25% of all notifications), distribution was restricted to the notifying country (16%), or distribution on the market was possible (14%). The decision on risk was usually not made (71% of all notifications). The product actions were diverse and included re-dispatching (22% of all notifications), withdrawal from the market (16%), or destruction (8%).

The open-source RASFF database can be an important tool in shaping public health within disease prevention (caused by heavy metals), life extension, and promoting physical health. Data on heavy metals can be used by authorities and non-governmental organizations, but also can contribute to raising consumer awareness of the occurrence of heavy metals. The EU authorities (e.g., European Food Safety Authority, Food and Veterinary Office) and national authorities can use these data in planning food controls and audits. In the long run, the RASFF data can affect change or amend the European food law (regulations or directives). Particular attention (within product categories and origin) should be paid to alert notifications, because in these cases the health risk has a potential to be serious. It appears that border rejections account for the limited number of alert notifications, because the number of alerts significantly decreased after introducing border rejections in 2008. However, in the RASFF database data on year, (last) origin country, heavy metal name, (average) concentration of heavy metal are scattered. They should be unambiguously given in separate columns. It should be also indicated if the risk was serious or not serious (now the risk is often identified as “undecided”). This way of presenting the data would allow to follow the trends of occurrence of heavy metals and focus on the most important problems.

## Figures and Tables

**Table 1:** Numbers of notifications of heavy metals within food.

Heavy Metal	Mercury (1203)	Cadmium (741)	Lead (215)
**Year**	2007 (125), 2014 (117), 2015 (106), 2013 (104), 2016 (100), 2009 (92), 2008 (84), 2010 (82), 2011 (81), 2012 (74), 2006 (69)	2003 (103), 2006 (75), 2009 (65), 2005 (63), 2007 (58), 2004 (47), 2011 (45), 2010 (44), 2014 (40), 2008 (39), 2016 (37)	2005 (30), 2006 (27), 2003 (20), 2007 (18), 2008 (16), 2012 (13), 2015 (13), 2011 (12)
**Product category**	fish and fish products (1135)	fish and fish products (160), cephalopods and products thereof (151), crustaceans and products thereof (143), molluscs and products thereof (obsolete) (114), fruits and vegetables (54)	fruits and vegetables (47), dietetic foods, food supplements, fortified foods (44), food contact materials (35), cocoa and cocoa preparations, coffee and tea (14), meat and meat products (other than poultry) (14), confectionery (13)
**Notifying country**	Italy (648), Spain (133), France (125), Germany (94)	Italy (264), Spain (137), Greece (49), Germany (47), France (45), United Kingdom (32), Portugal (27)	Italy (53), Germany (21), Poland (20), United Kingdom (18), Belgium (16), Lithuania (11)
**Origin country**	Spain (409), Vietnam (88), Indonesia (72), Portugal (57), France (29), Chile (28), Sri Lanka (28), Denmark (27), Brazil (26), Namibia (26), Senegal (24), Ecuador (22), Taiwan (22), Tunisia (22), China (21), Singapore (20), Panama (19), India (18), New Zealand (18)	Thailand (76), India (73), France (63), Singapore (61), China (44), Australia (39), Vietnam (29), Chile (28), Indonesia (28), Morocco (24), Italy (19), Spain (19), Argentina (17), Poland (17), Falkland Islands (16), Ireland (13), Yemen (13), United Kingdom (12)	China (42), India (15), Ukraine (12), Italy (11), United States (11), Germany (10), Netherlands (10), Turkey (10), Brazil (9), Nigeria (8), Greece (7), France (6)
**Notification basis**	official control on the market (706), border control—consignment detained (226), border control—consignment released (173)	border control—consignment detained (289), official control on the market (247)	official control on the market (101), border control—consignment detained (83)
**Notification type**	alert (510), information (348)	information (433)	information (95), alert (72)
**Distribution status**	distribution restricted to notifying country (244), distribution on the market (possible) (208), no distribution (160), (not specified) (105), product (presumably) no longer on the market (94), distribution to other member countries (87)	(not specified) (190), no distribution (163), distribution on the market (possible) (128), distribution restricted to notifying country (116)	no distribution (63), (not specified) (40), distribution on the market (possible) (37), distribution restricted to notifying country (29)
**Risk decision**	undecided (749), serious (442)	undecided (619)	undecided (172)
**Action taken**	withdrawal from the market (218), re-dispatch (148), destruction (128), (not specified) (116), official detention (116), seizure (87), import not authorized (58), informing authorities (58)	re-dispatch (188), withdrawal from the market (96), (not specified) (72), import not authorized (69), destruction (67), product recall or withdrawal (31)	re-dispatch (54), withdrawal from the market (35), destruction (26), product recall or withdrawal (15), (not specified) (14), official detention (12)

**Table 2:** Numbers of notifications of heavy metals within food contact material.

Heavy Metal	Chromium (387)	Cadmium (139)	Nickel (124)
**Year**	2013 (64), 2011 (58), 2010 (53), 2012 (49), 2014 (42), 2015 (38), 2009 (35)	2011 (27), 2012 (24), 2016 (17), 2010 (15), 2008 (14), 2013 (14)	2013 (27), 2010 (17), 2012 (16), 2014 (15), 2015 (11)
**Product category**	food contact materials (387)	food contact materials (139)	food contact materials (124)
**Notifying country**	Italy (342)	Poland (45), Germany (27), Czech Republic (19), Finland (14)	Italy (106)
**Origin country**	China (330), Hong Kong (25)	China (93)	China (88)
**Notification basis**	border control—consignment detained (304)	official control on the market (111)	border control—consignment detained (103)
**Notification type**	border rejection (319)	alert (56), information for attention (42)	border rejection (109)
**Distribution status**	no distribution (201), product not (yet) placed on the market (102)	distribution restricted to notifying country (49), distribution to other member countries (27), distribution on the market (possible) (19)	no distribution (58), product not (yet) placed on the market (44)
**Risk decision**	undecided (259)	undecided (81), serious (55)	undecided (67), not serious (53)
**Action taken**	re-dispatch (147), re-dispatch or destruction (43), import not authorized (34), official detention (34), return to consignor (29), withdrawal from the market (29)	withdrawal from the market (72), recall from consumers (16), official detention (12)	re-dispatch (55), import not authorized (18), official detention (10), placed under customs seals (10)

**Table 3:** Numbers of notifications of heavy metals within feed.

Heavy Metal	Arsenic (47)	Cadmium (34)	Mercury (30)	Lead (29)
**Year**	2012 (16), 2011 (16)	2009 (4), 2013 (4), 2014 (4)	2016 (9), 2013 (5), 2015 (5)	2016 (5), 2002 (4), 2006 (3), 2013 (3)
**Product category**	feed materials (18), pet food (12)	feed additives (10), feed materials (9), animal nutrition (obsolete) (7)	feed materials (13), pet food (9)	animal nutrition (obsolete) (8), feed materials (7), feed additives (6), feed for food-producing animals (obsolete) (5)
**Notifying country**	Italy (10), Germany (8), Belgium (7), Austria (5), United Kingdom (5)	Belgium (8), Germany (4), Norway (4)	Italy (14), Spain (5), Germany (4)	Netherlands (6), Germany (5), Belgium (4)
**Origin country**	Thailand (11), Poland (5)	China (8), Spain (4)	Thailand (10), Russia (5), Spain (4)	Netherlands (5), Spain (4), Turkey (3)
**Notification basis**	official control on the market (21), border control—consignment detained (12), company’s own check (11)	company’s own check (14), official control on the market (14)	official control on the market (14), border control—consignment detained (12)	company’s own check (12), official control on the market (10)
**Notification type**	alert (13), border rejection (12), information for follow-up (11)	information (12), information for follow-up (11)	border rejection (12), information for follow-up (10)	alert (9), information for follow-up (9), information (8)
**Distribution status**	no distribution (11), distribution restricted to notifying country (8), distribution to other member countries (8), information on distribution not (yet) available (7)	distribution on the market (possible) (10), distribution to other member countries (10), distribution restricted to notifying country (8)	product not (yet) placed on the market (11), distribution restricted to notifying country (7), no distribution from notifying country (6)	(not specified) (7), distribution on the market (possible) (6), distribution to other member countries (6), distribution restricted to notifying country (5)
**Risk decision**	undecided (37)	undecided (25)	not serious (18)	undecided (21)
**Action taken**	re-dispatch (8), withdrawal from the market (7), destruction (4), informing recipients (4), official detention (4)	withdrawal from the market (7), (not specified) (4), official detention (4), seizure (3)	re-dispatch (6), import not authorized (5), official detention (5), withdrawal from the market (5)	(not specified) (7), return to consignor (4), withdrawal from the market (4)

## Supplementary Materials

The following are available online at http://www.mdpi.com/1660-4601/15/2/365/s1, Figure S1: Similarities of RASFF notifications on heavy metals and year within food: (a) joining; (b) two-way joining, Figure S2: Similarities of RASFF notifications on heavy metals and product category within food: (a) joining; (b) two-way joining, Figure S3: Similarities of RASFF notifications on heavy metals and notifying country within food: (a) joining; (b) two-way joining, Figure S4: Similarities of RASFF notifications on heavy metals and origin country within food: (a) joining; (b) two-way joining, Figure S5: Similarities of RASFF notifications on heavy metals and notification basis within food: (a) joining; (b) two-way joining, Figure S6: Similarities of RASFF notifications on heavy metals and notification type within food: (a) joining; (b) two-way joining, Figure S7: Similarities of RASFF notifications on heavy metals and distribution status within food: (a) joining; (b) two-way joining, Figure S8: Similarities of RASFF notifications on heavy metals and risk decision within food: (a) joining; (b) two-way joining, Figure S9: Similarities of RASFF notifications on heavy metals and action taken within food: (a) joining; (b) two-way joining, Figure S10: Similarities of RASFF notifications on heavy metals and year within food contact material: (a) joining; (b) two-way joining, Figure S11: Similarities of RASFF notifications on heavy metals and notifying country within food contact material: (a) joining; (b) two-way joining, Figure S12: Similarities of RASFF notifications on heavy metals and origin country within food contact material: (a) joining; (b) two-way joining, Figure S13: Similarities of RASFF notifications on heavy metals and notification basis within food contact material: (a) joining; (b) two-way joining, Figure S14: Similarities of RASFF notifications on heavy metals and notification type within food contact material: (a) joining; (b) two-way joining, Figure S15: Similarities of RASFF notifications on heavy metals and distribution status within food contact material: (a) joining; (b) two-way joining, Figure S16: Similarities of RASFF notifications on heavy metals and risk decision within food contact material: (a) joining; (b) two-way joining, Figure S17: Similarities of RASFF notifications on heavy metals and action taken within food contact material: (a) joining; (b) two-way joining, Figure S18: Similarities of RASFF notifications on heavy metals and year within food: (a) joining; (b) two-way joining, Figure S19: Similarities of RASFF notifications on heavy metals and product category within feed: (a) joining; (b) two-way joining, Figure S20: Similarities of RASFF notifications on heavy metals and notifying country within feed: (a) joining; (b) two-way joining, Figure S21: Similarities of RASFF notifications on heavy metals and origin country within feed: (a) joining; (b) two-way joining, Figure S22: Similarities of RASFF notifications on heavy metals and notification basis within feed: (a) joining; (b) two-way joining, Figure S23: Similarities of RASFF notifications on heavy metals and notification type within feed: (a) joining; (b) two-way joining, Figure S24: Similarities of RASFF notifications on heavy metals and distribution status within feed: (a) joining; (b) two-way joining, Figure S25: Similarities of RASFF notifications on heavy metals and risk decision within feed: (a) joining; (b) two-way joining, Figure S26: Similarities of RASFF notifications on heavy metals and action taken within feed: (a) joining; (b) two-way joining.
