# Supplementary material for: Heavy Metals in Notifications of Rapid Alert System for Food and Feed
Source: Int J Environ Res Public Health. 2018 Feb 20;15(2):365. doi: 10.3390/ijerph15020365 (PMC5858434; doi:10.3390/ijerph15020365)
Supplement: Supplementary file 1 [file ijerph-15-00365-s001.pdf]

Supplementary Materials: Heavy Metals in Notifications of Rapid Alert System for Food and Feed

Marcin Pigłowski

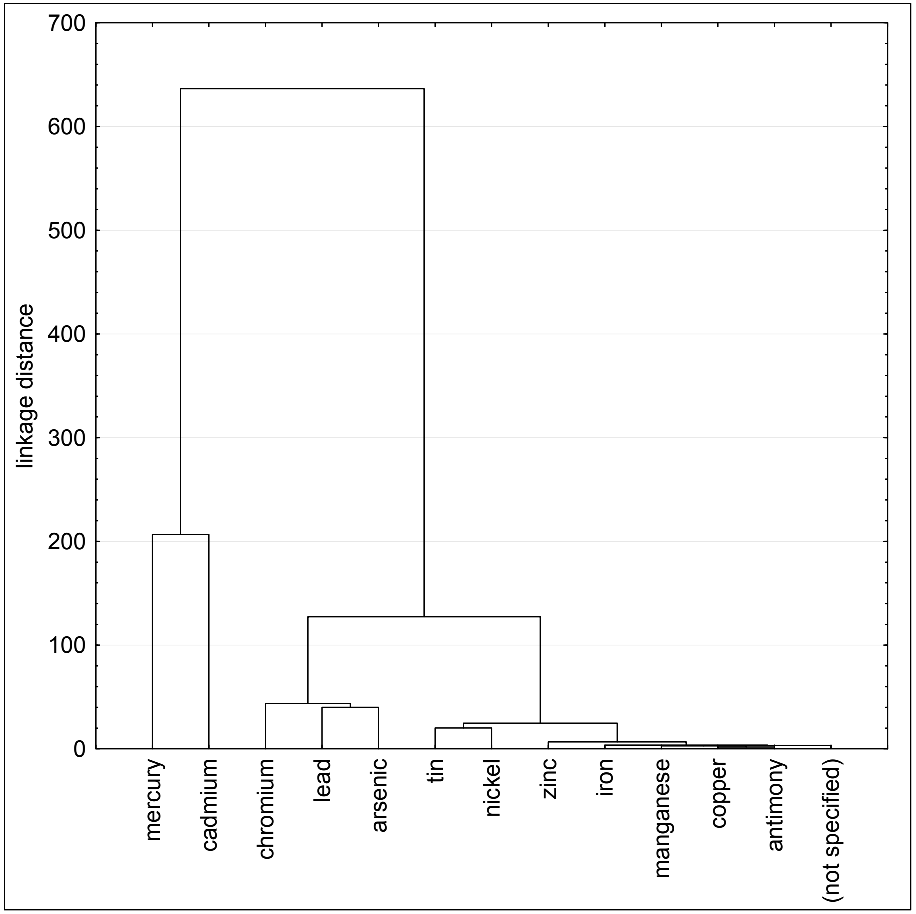

(a)

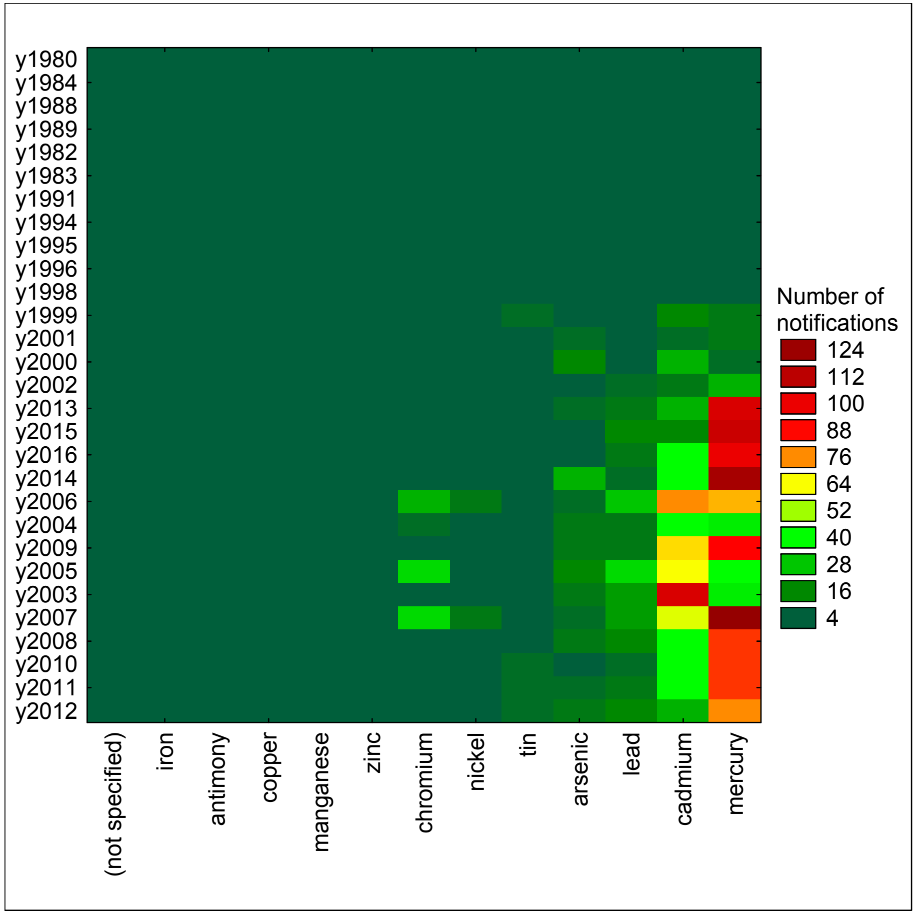

(b)

Figure S1: Similarities of RASFF notifications on heavy metals and year within food: (**a**) joining; (**b**) two-way joining.

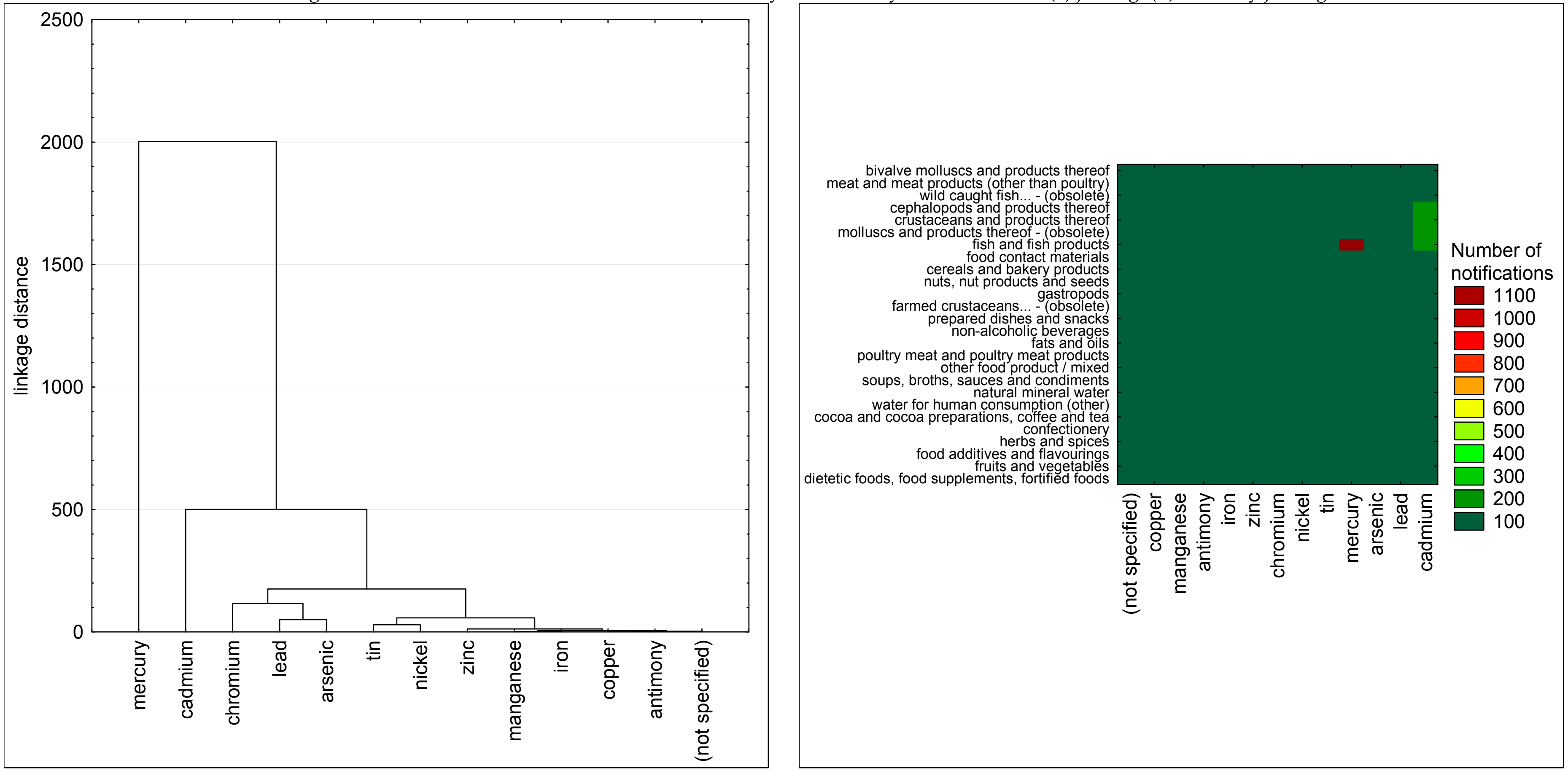

(**a**) (**b**)

Figure S2: Similarities of RASFF notifications on heavy metals and product category within food: (**a**) joining; (**b**) two-way joining.

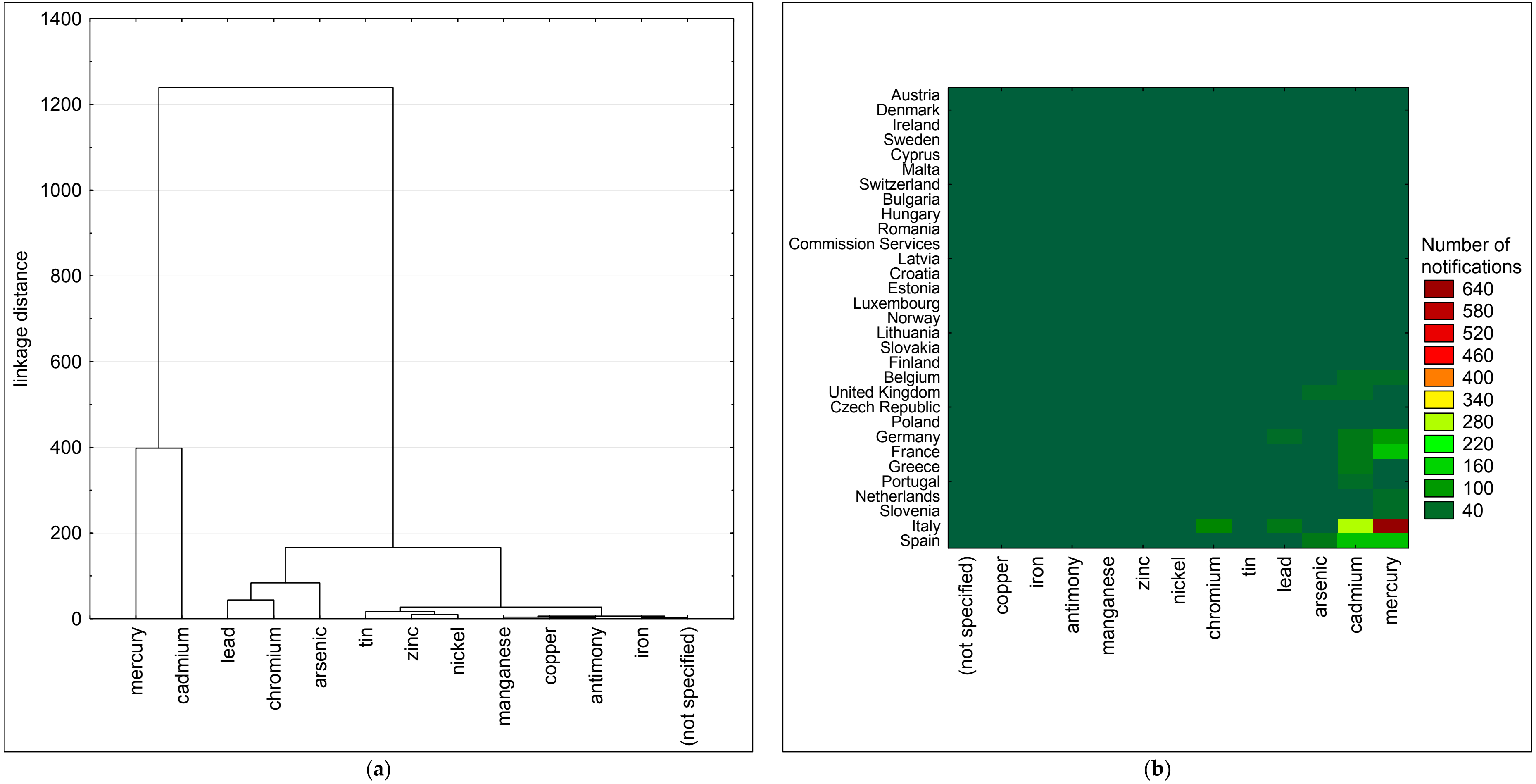

Figure S3: Similarities of RASFF notifications on heavy metals and notifying country within food: (**a**) joining; (**b**) two-way joining.

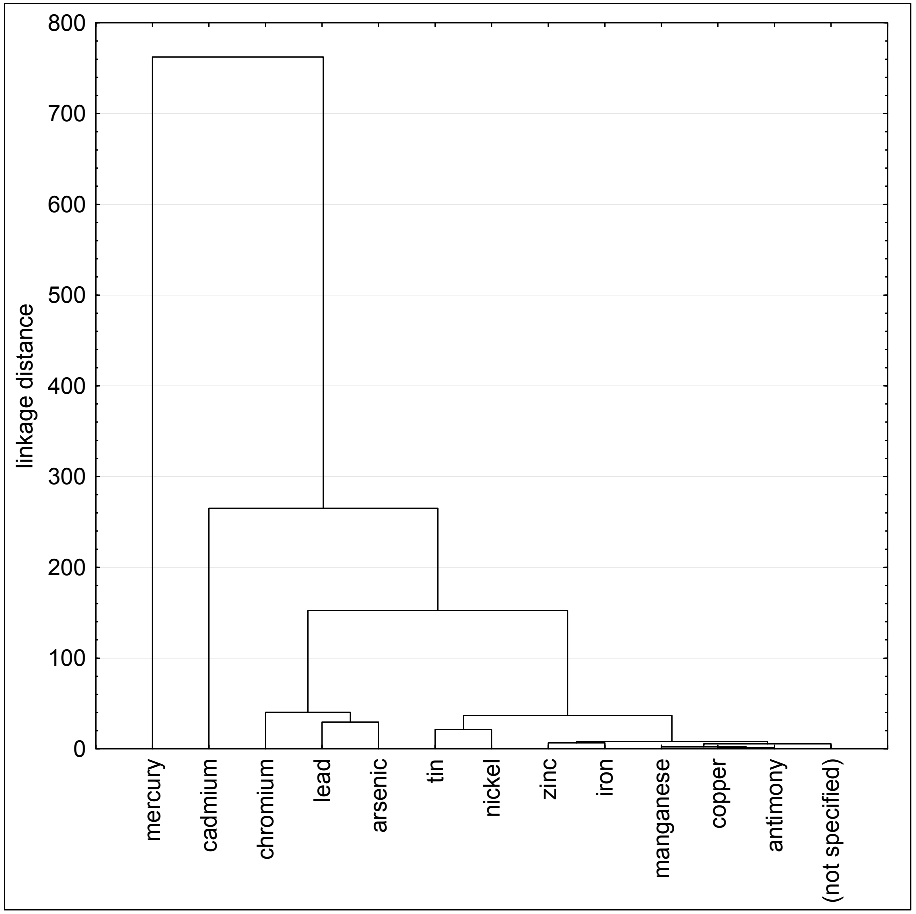

(**a**)

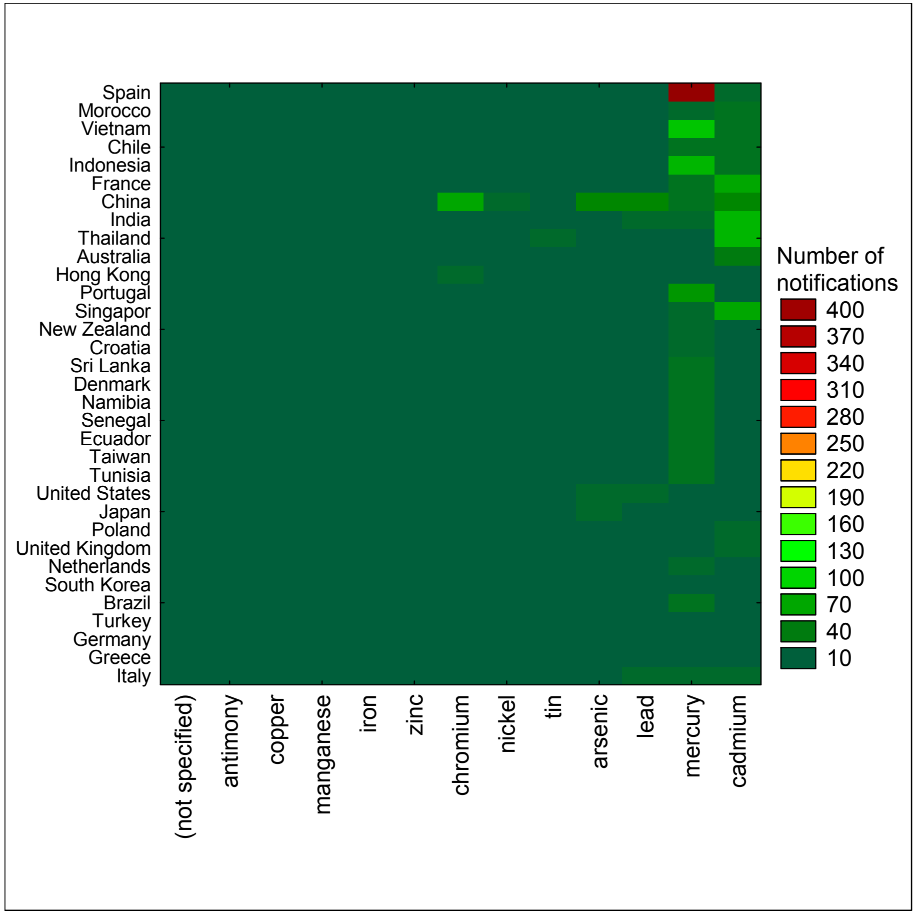

(**b**)

Figure S4: Similarities of RASFF notifications on heavy metals and origin country within food: (**a**) joining; (**b**) two-way joining.

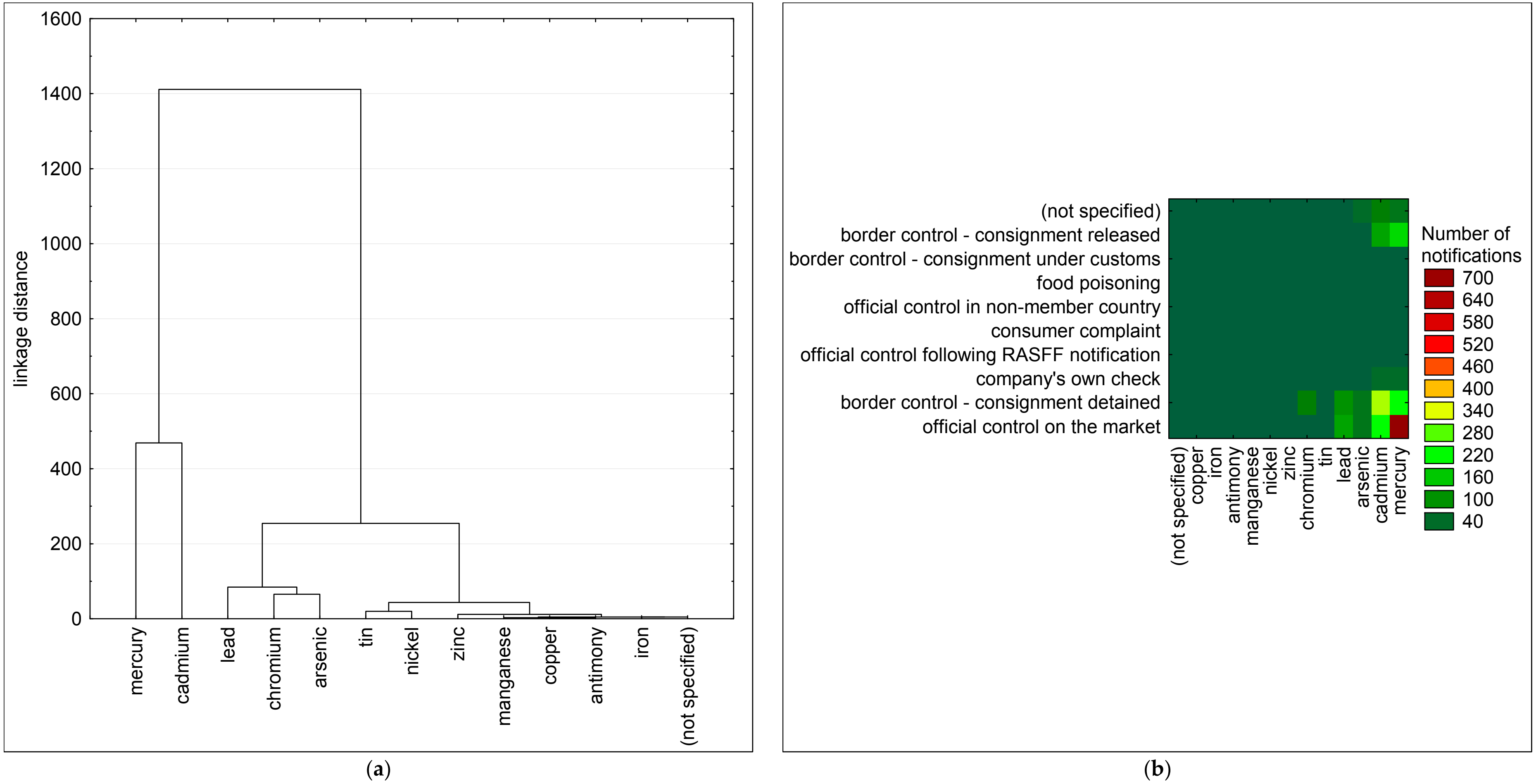

Figure S5: Similarities of RASFF notifications on heavy metals and notification basis within food: (**a**) joining; (**b**) two-way joining.

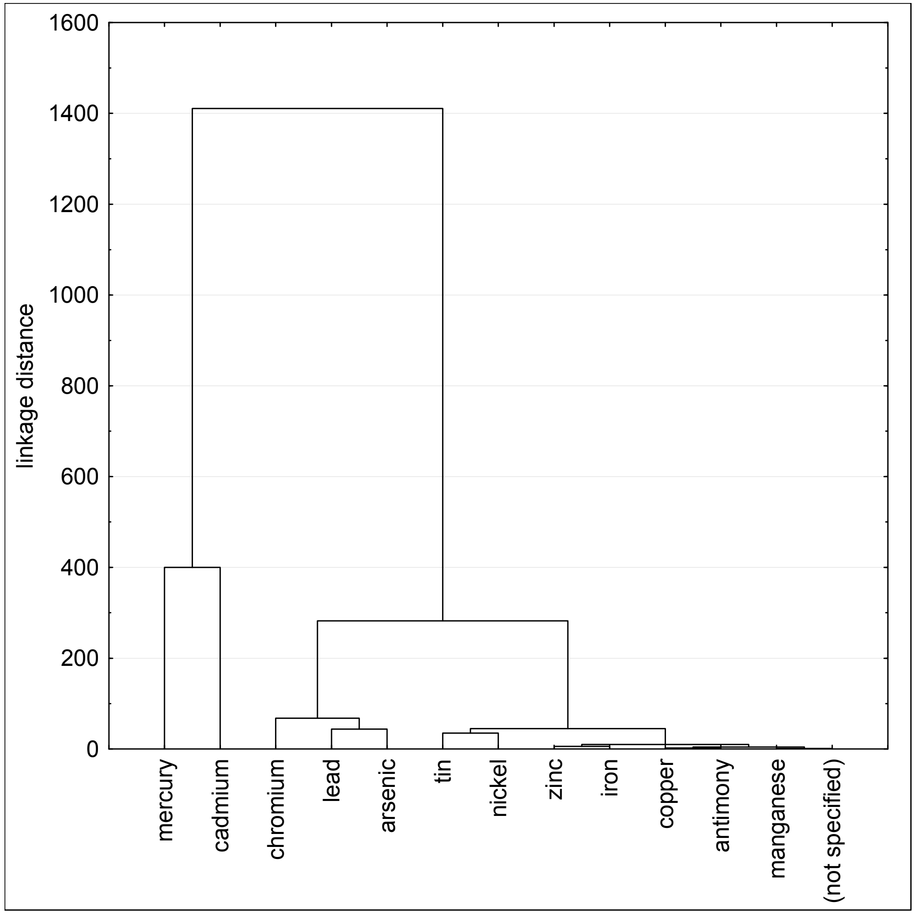

(**a**)

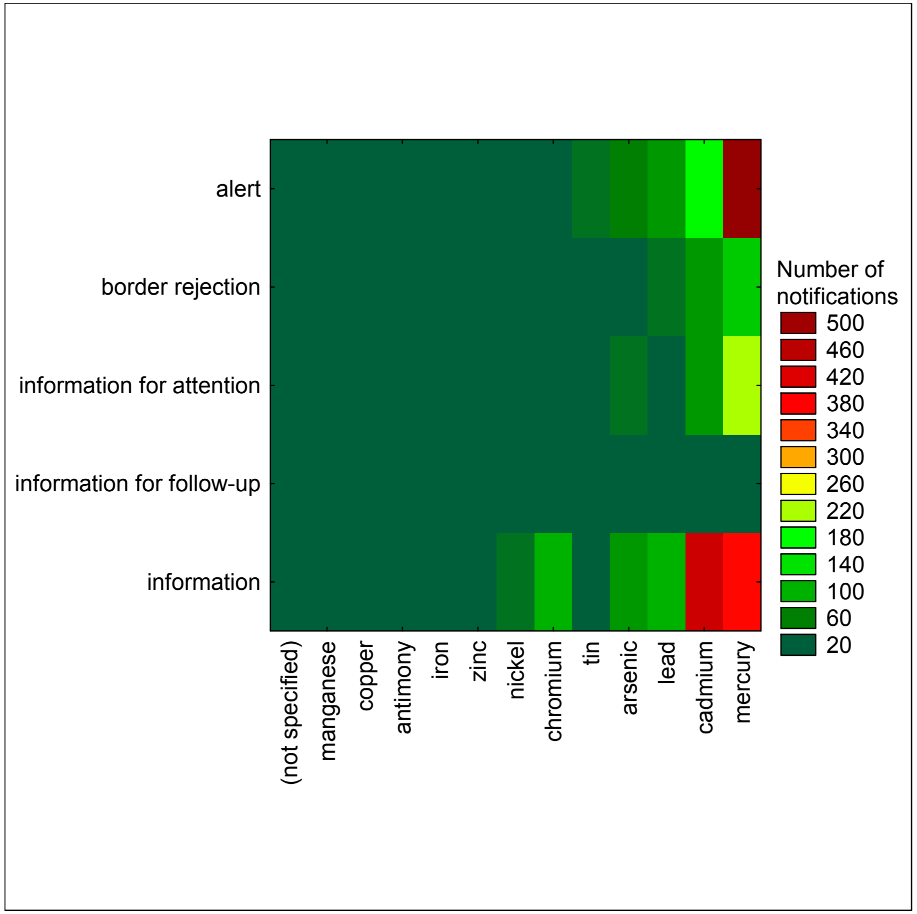

(**b**)

Figure S6: Similarities of RASFF notifications on heavy metals and notification type within food: (**a**) joining; (**b**) two-way joining.

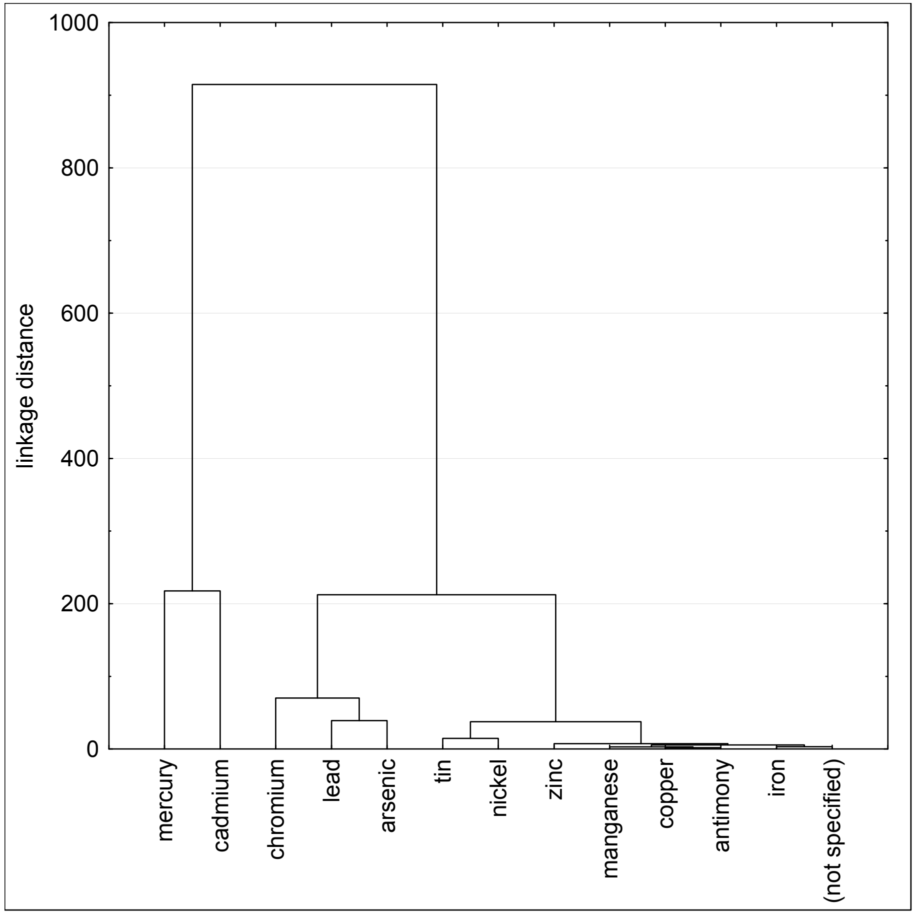

(**a**)

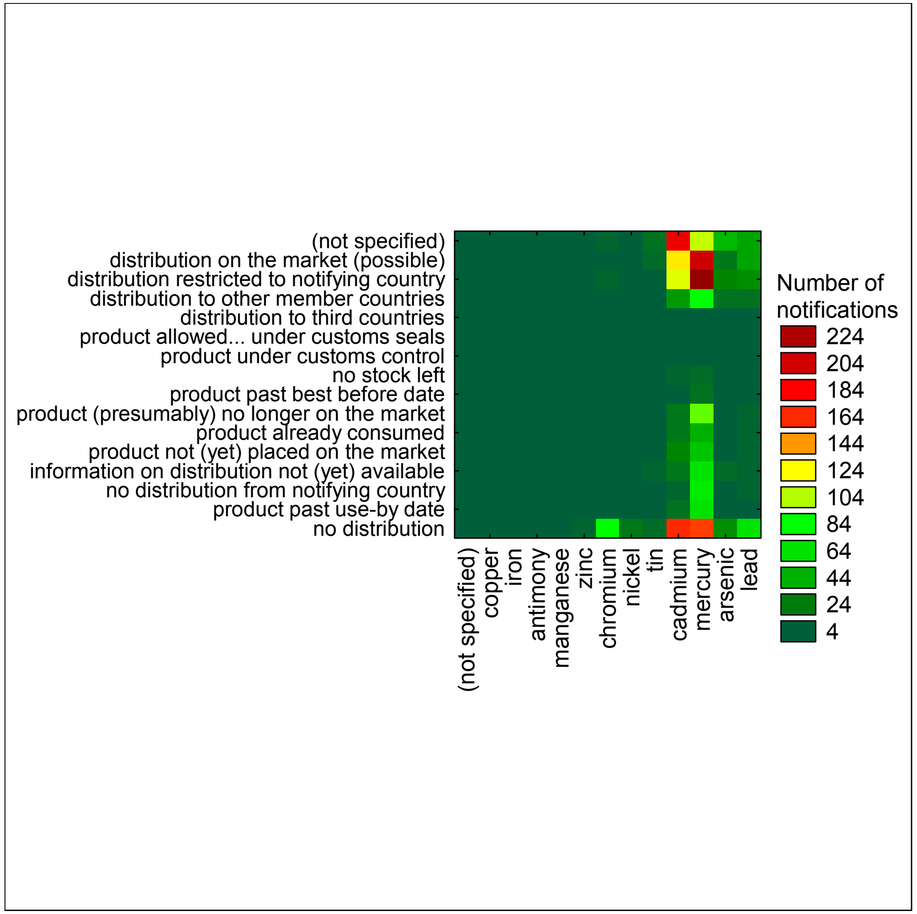

(**b**)

Figure S7: Similarities of RASFF notifications on heavy metals and distribution status within food: (**a**) joining; (**b**) two-way joining.

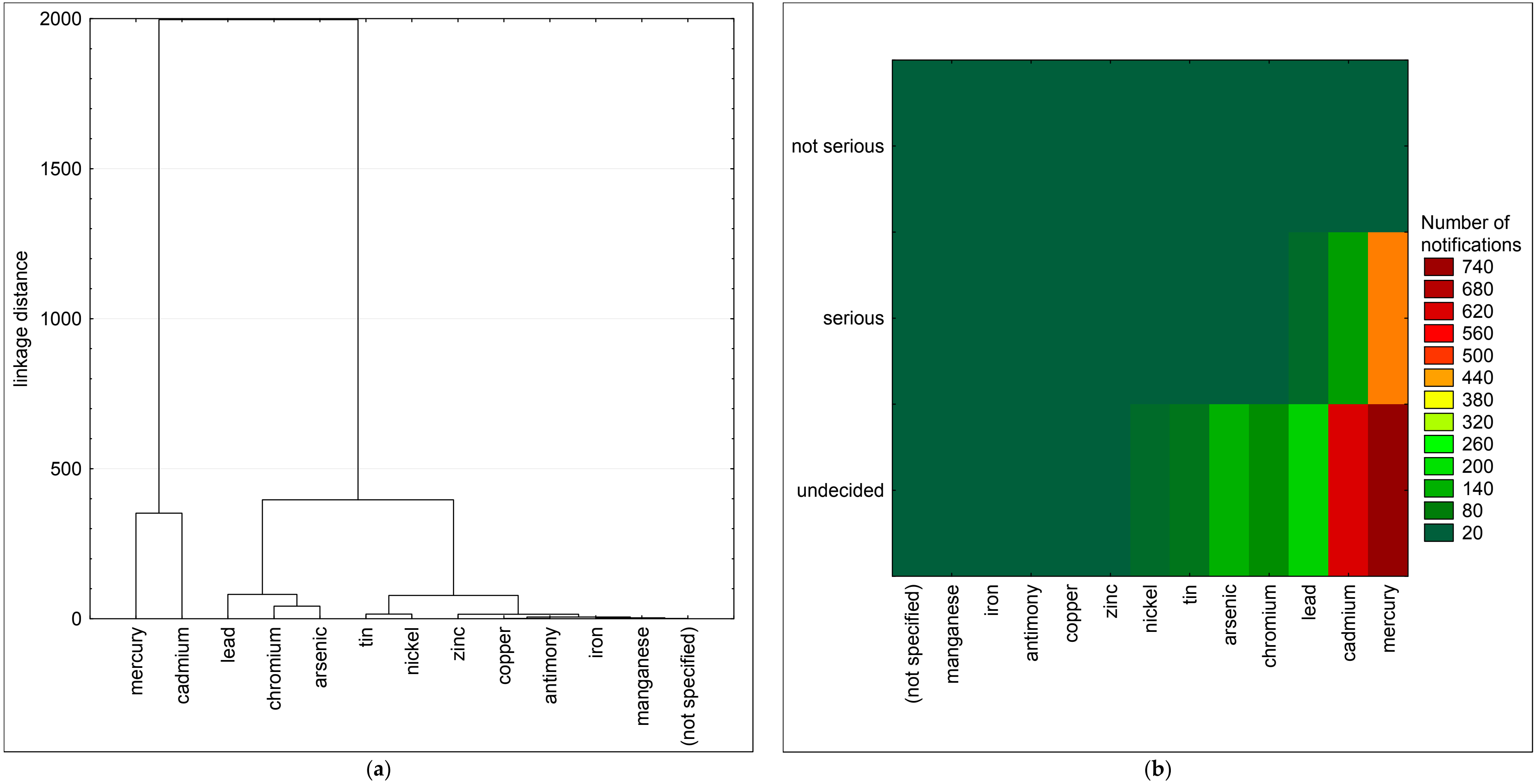

Figure S8: Similarities of RASFF notifications on heavy metals and risk decision within food: (**a**) joining; (**b**) two-way joining.

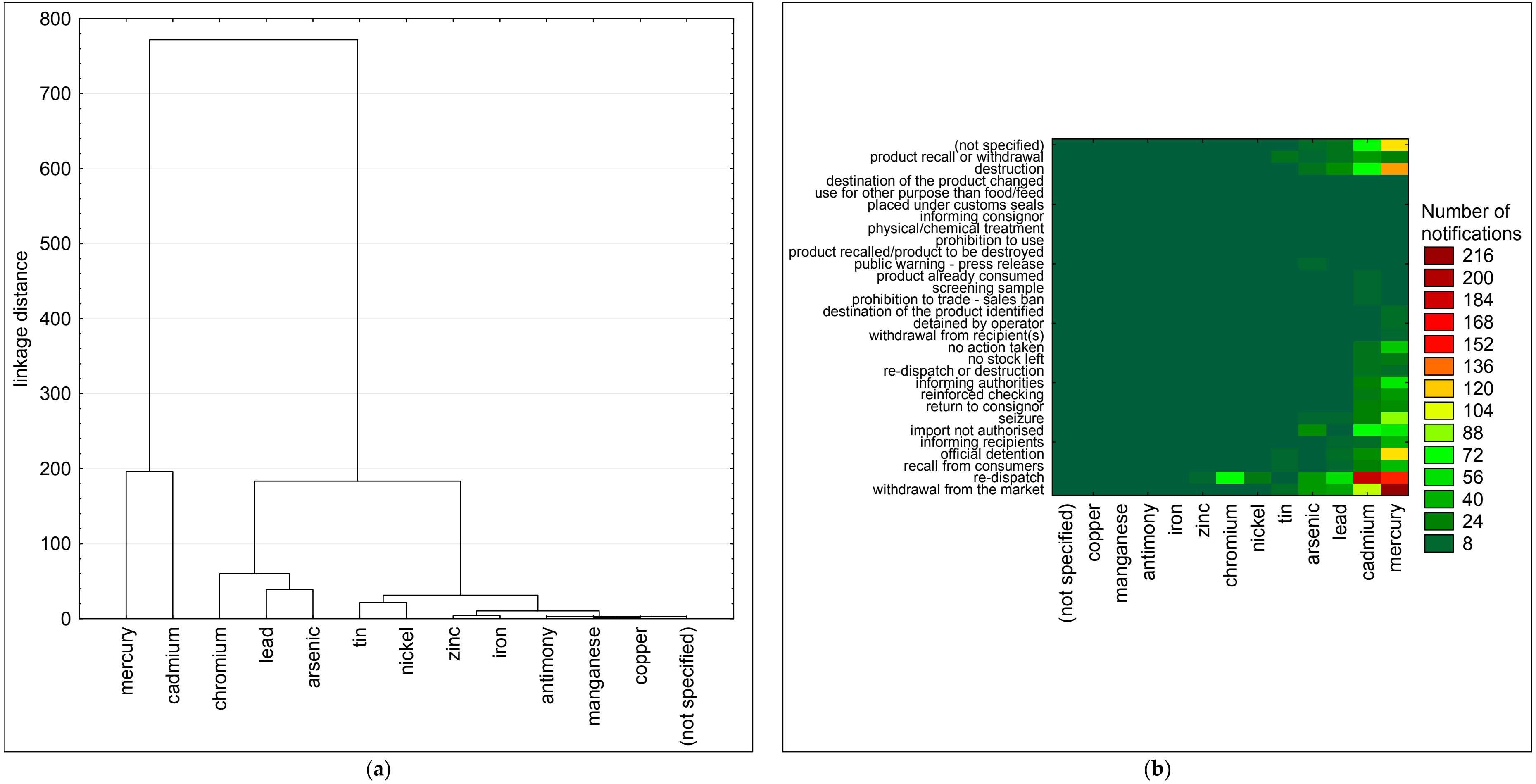

Figure S9: Similarities of RASFF notifications on heavy metals and action taken within food: (**a**) joining; (**b**) two-way joining.

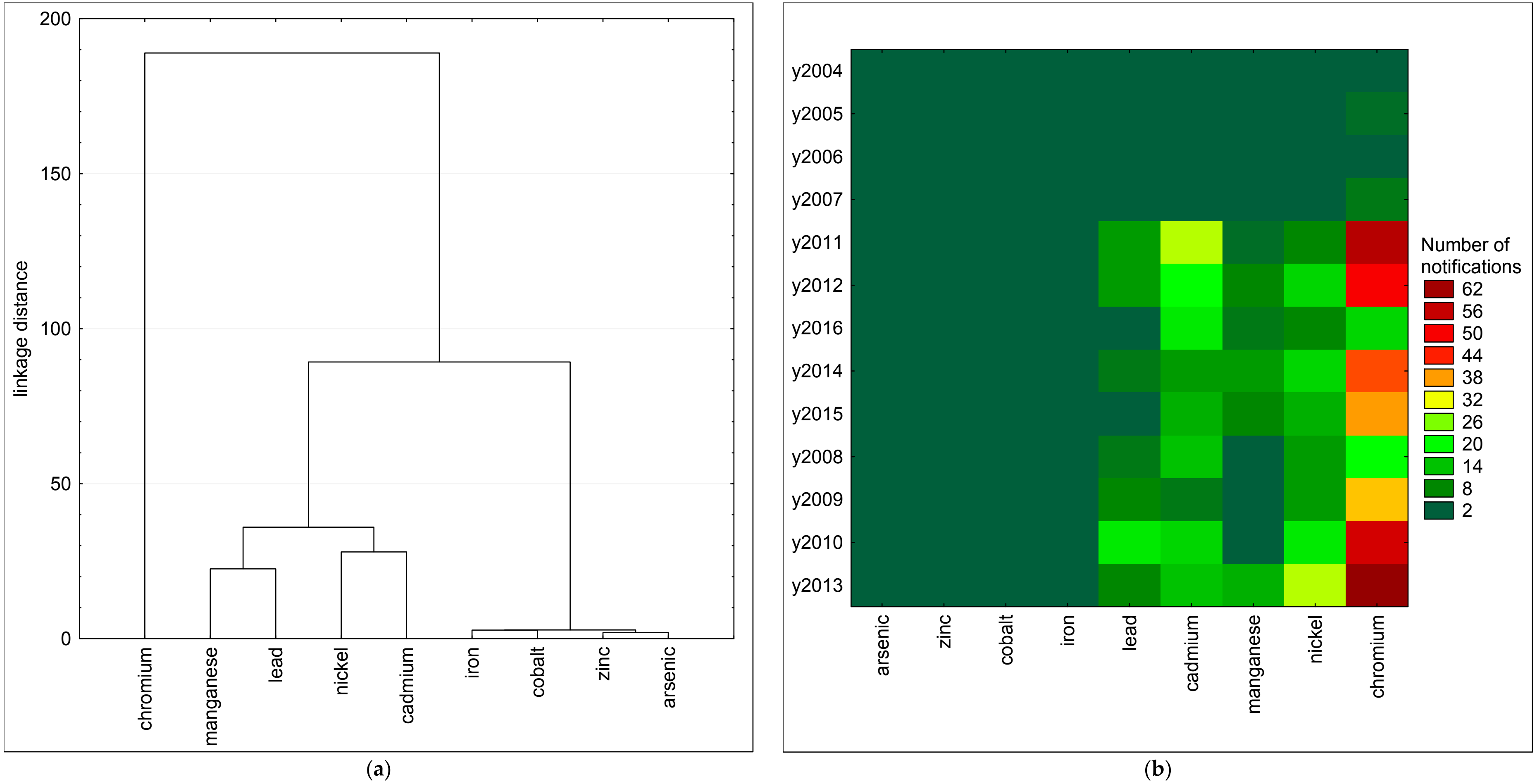

Figure S10: Similarities of RASFF notifications on heavy metals and year within food contact material: (**a**) joining; (**b**) two-way joining.

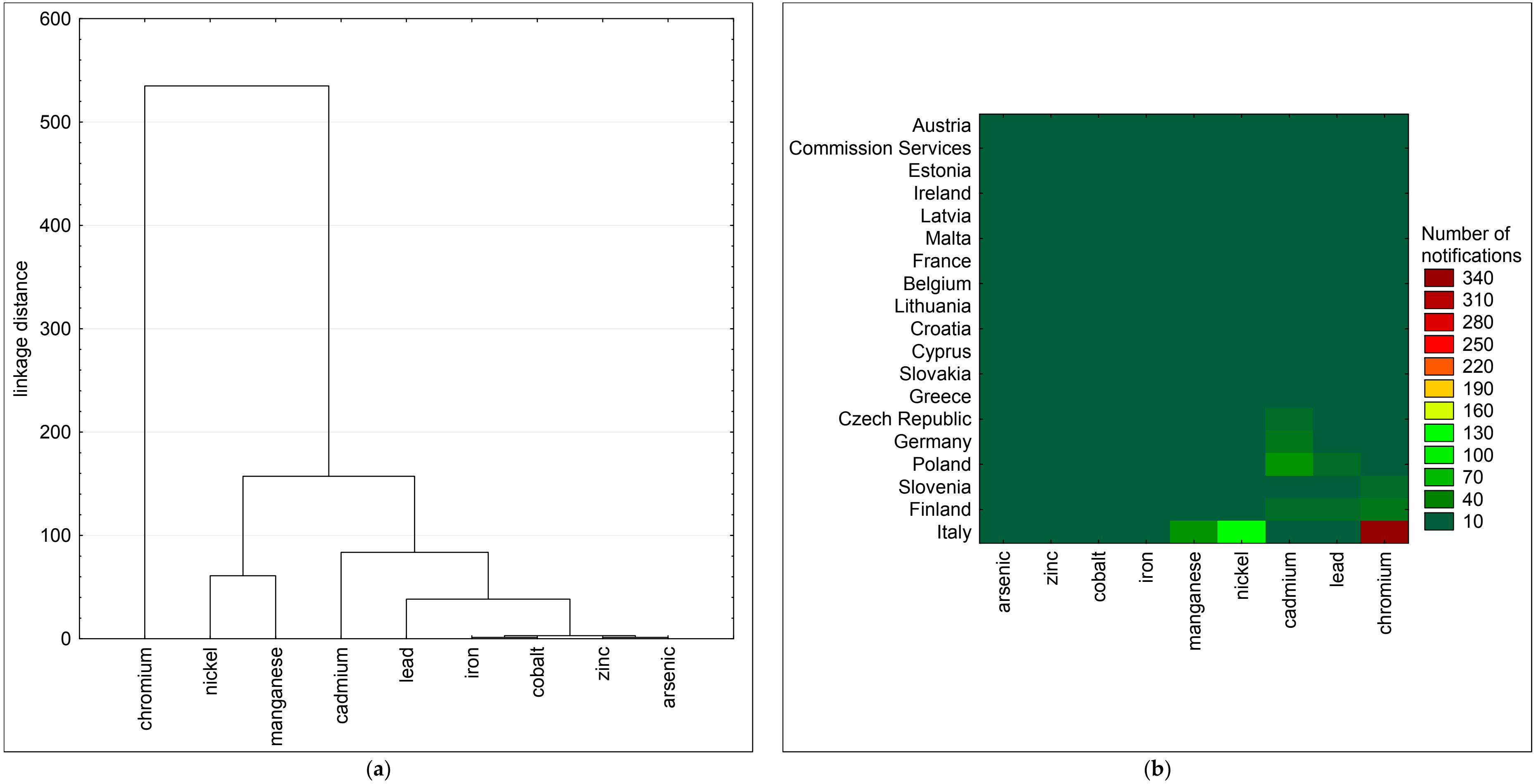

Figure S11: Similarities of RASFF notifications on heavy metals and notifying country within food contact material: (**a**) joining; (**b**) two-way joining.

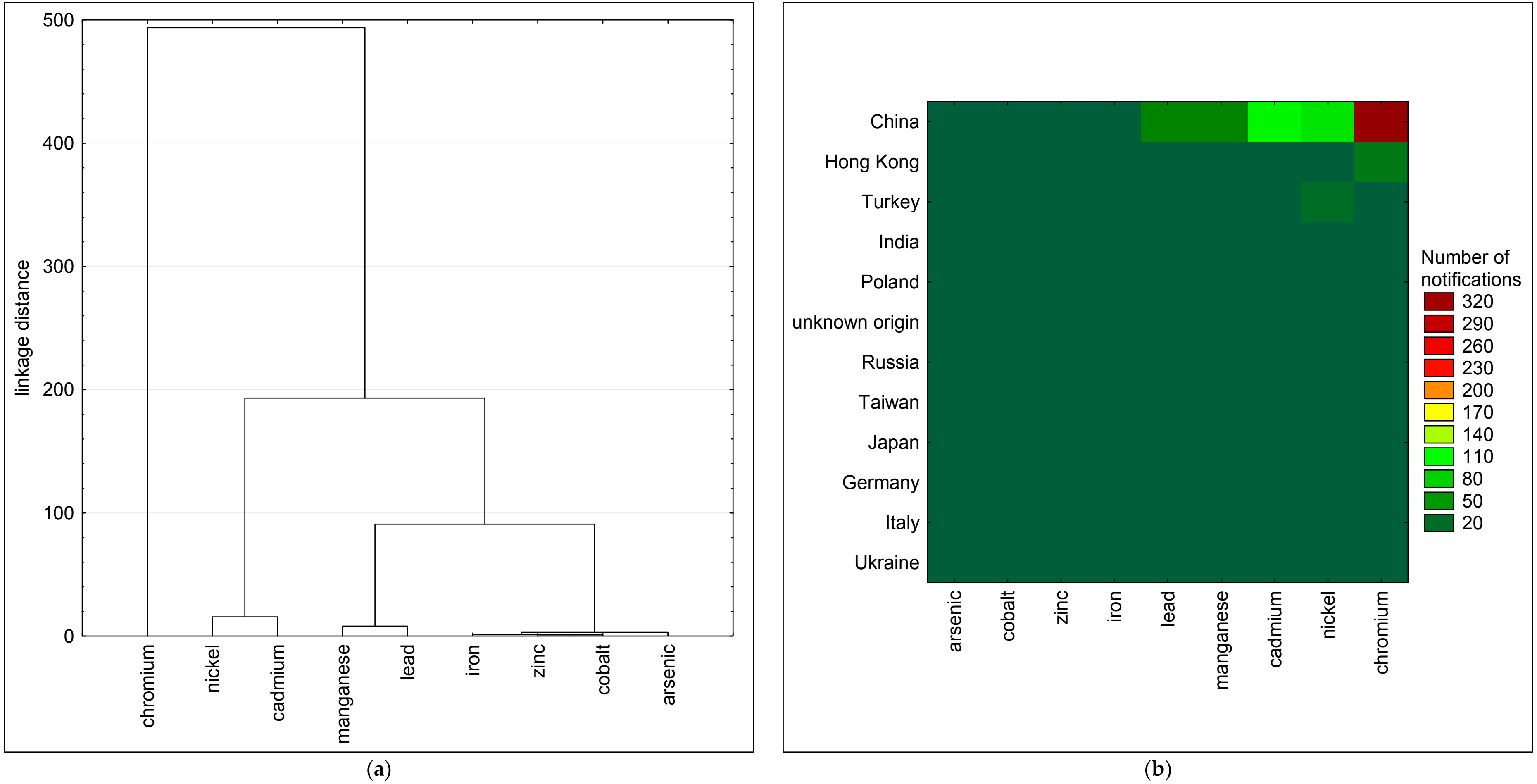

Figure S12: Similarities of RASFF notifications on heavy metals and origin country within food contact material: (**a**) joining; (**b**) two-way joining.

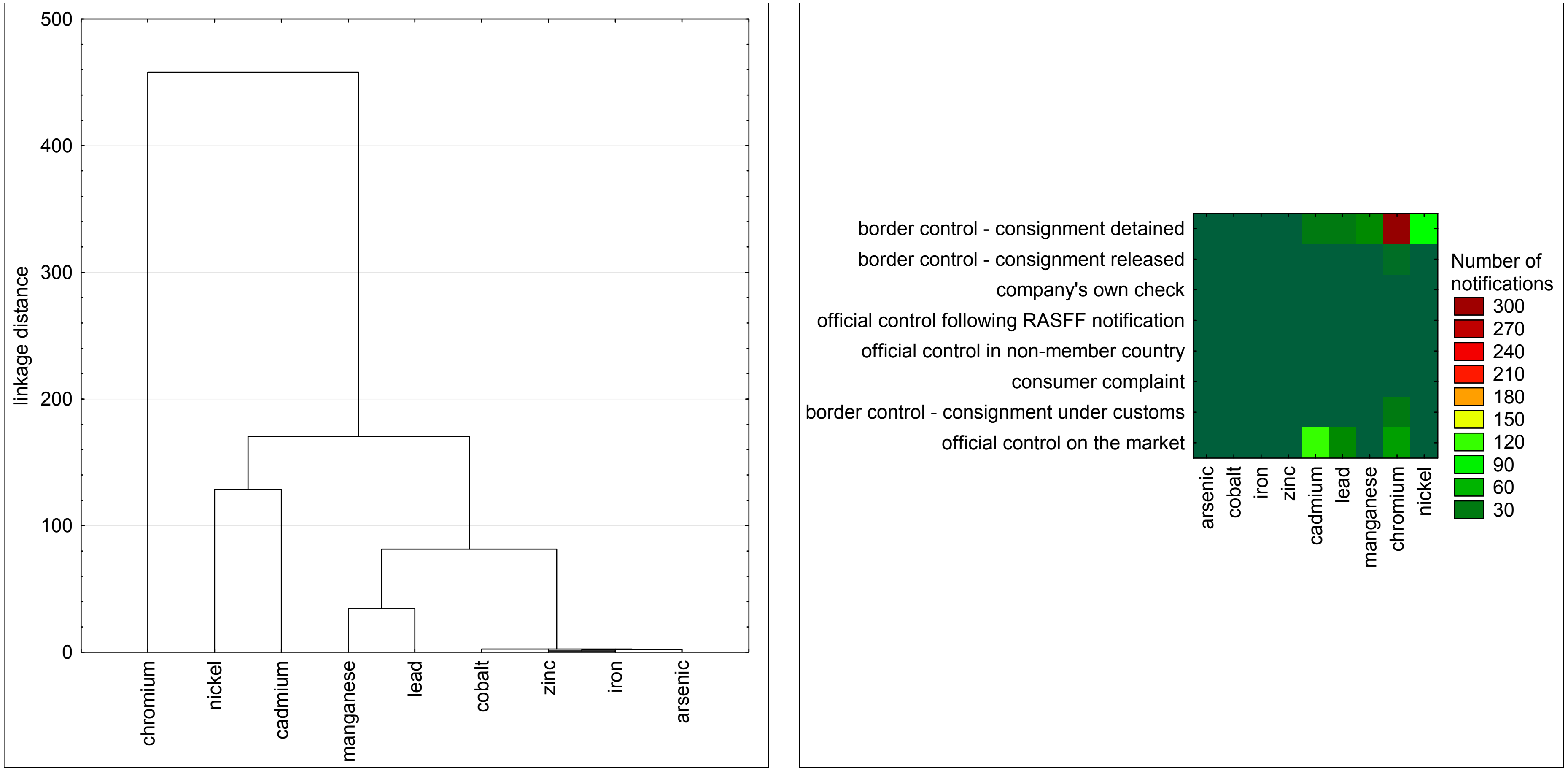

(**a**) (**b**)

Figure S13: Similarities of RASFF notifications on heavy metals and notification basis within food contact material: (**a**) joining; (**b**) two-way joining.

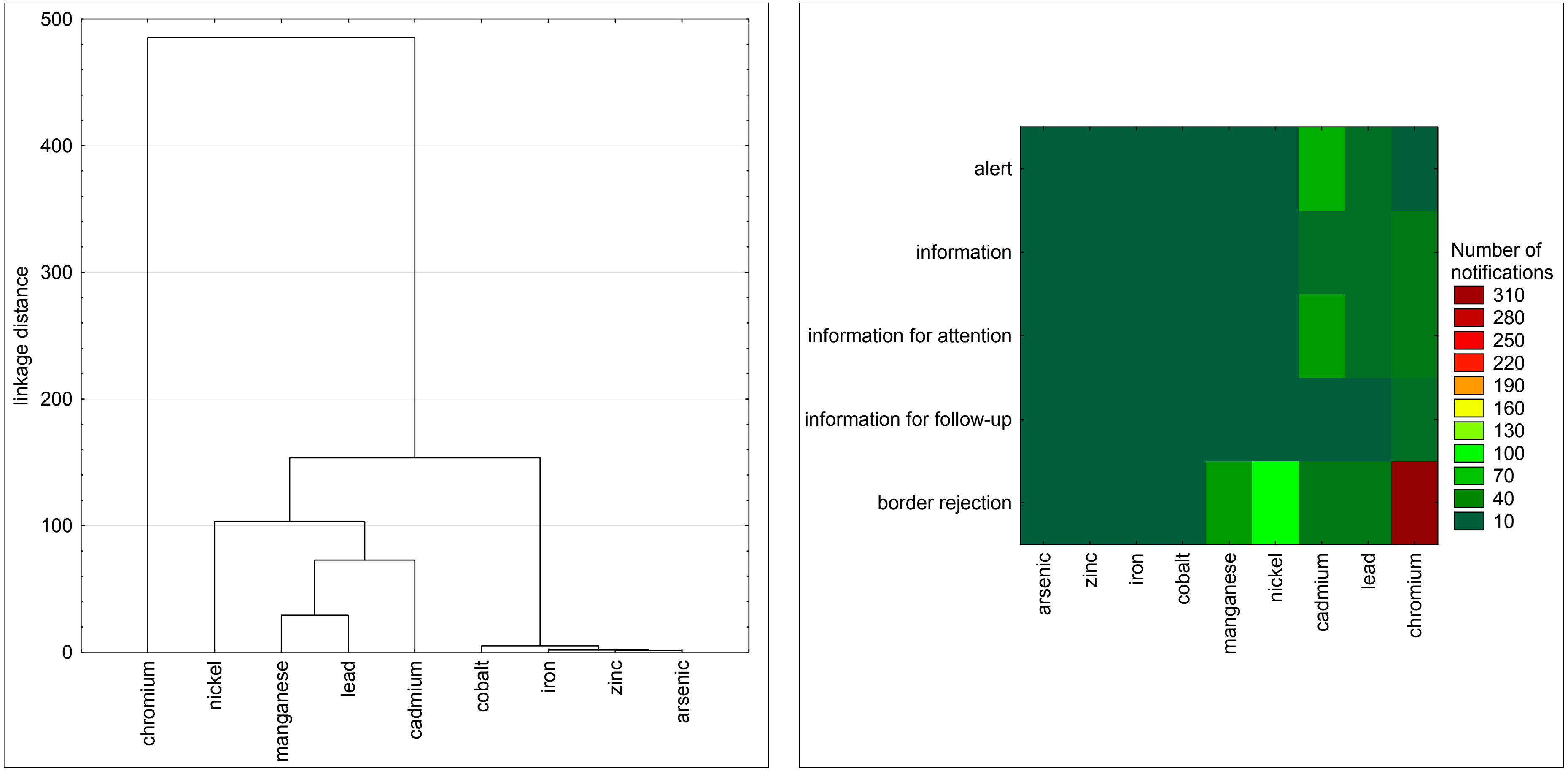

(**a**) (**b**)

Figure S14: Similarities of RASFF notifications on heavy metals and notification type within food contact material: (**a**) joining; (**b**) two-way joining.

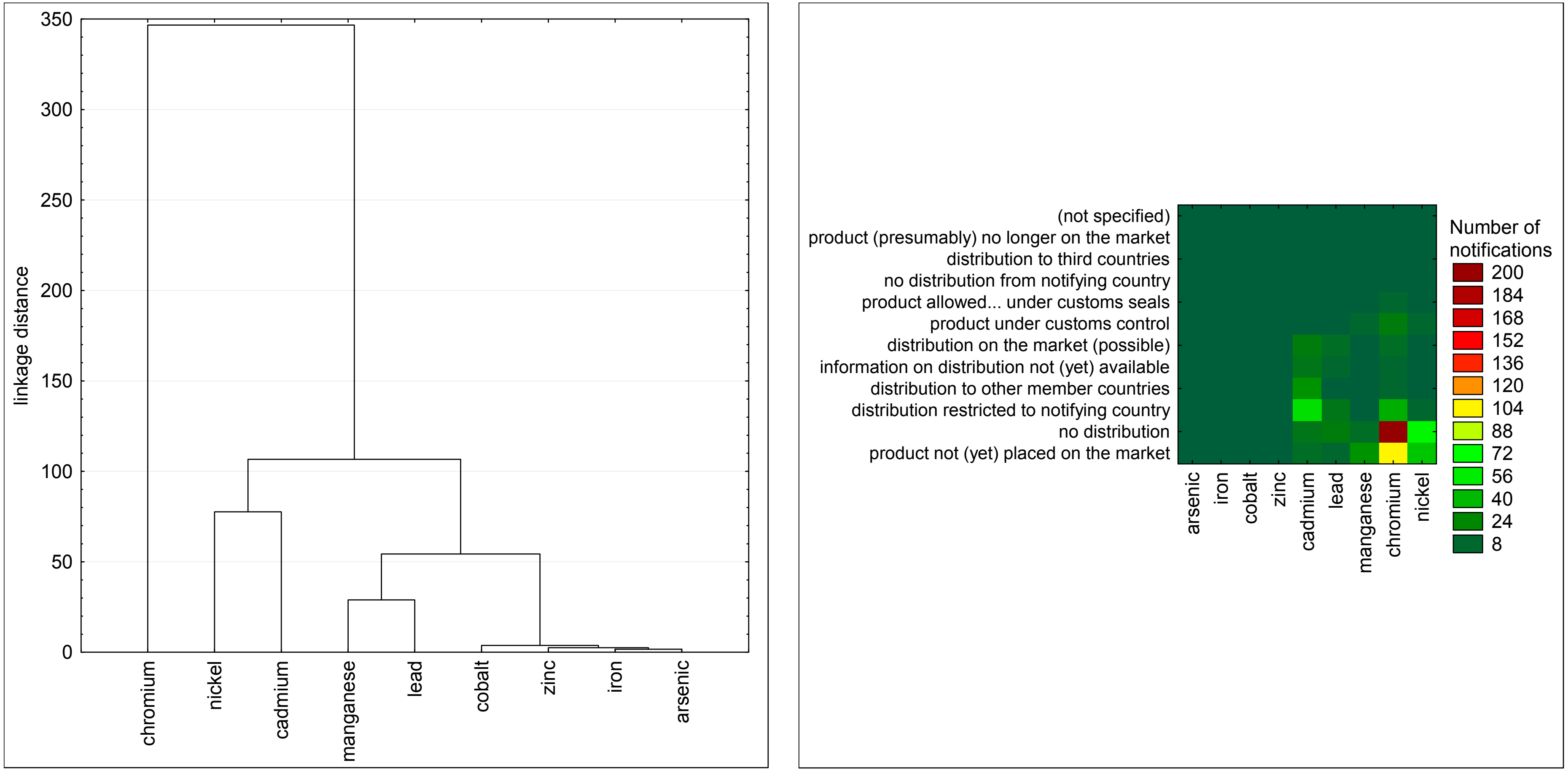

(**a**) (**b**)

Figure S15: Similarities of RASFF notifications on heavy metals and distribution status within food contact material: (**a**) joining; (**b**) two-way joining.

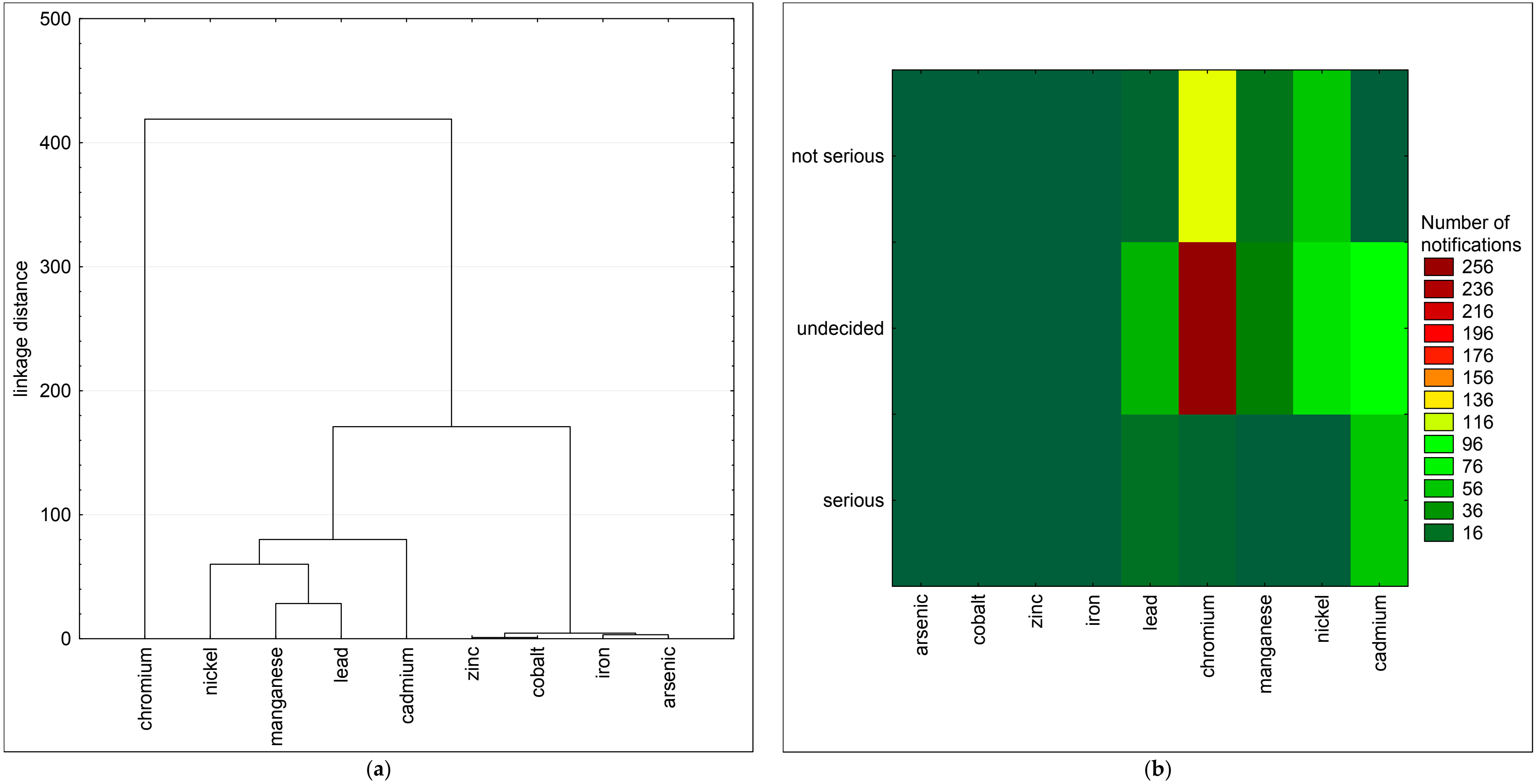

Figure S16: Similarities of RASFF notifications on heavy metals and risk decision within food contact material: (**a**) joining; (**b**) two-way joining.

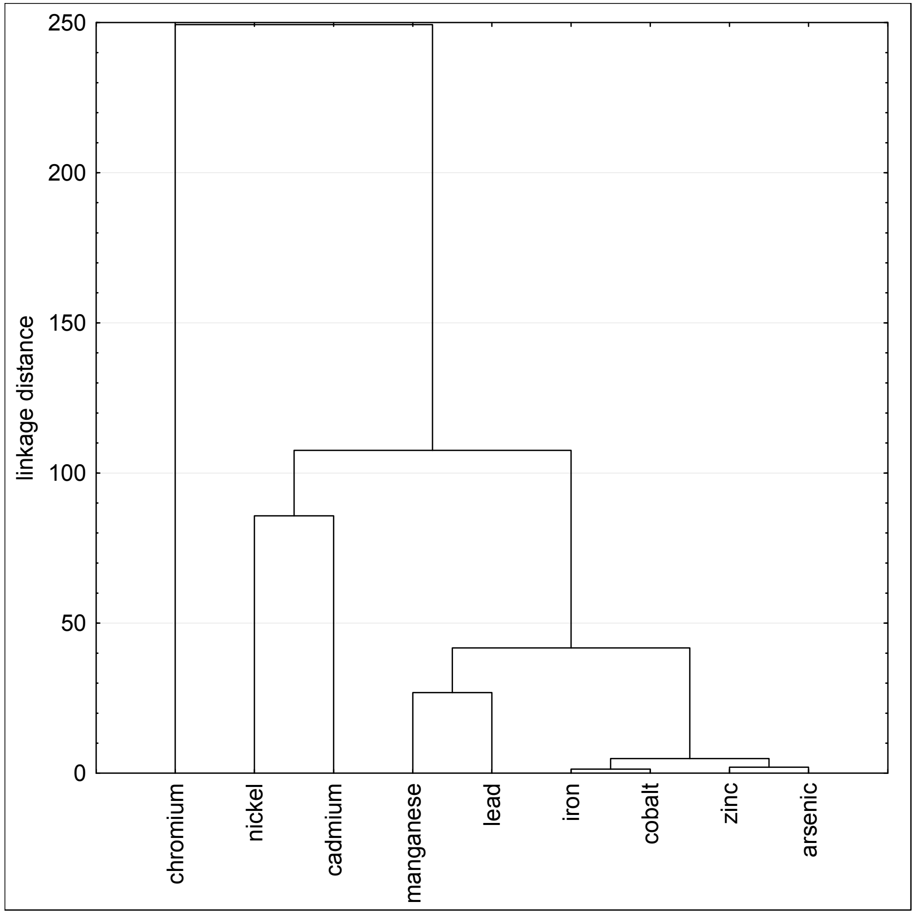

(**a**)

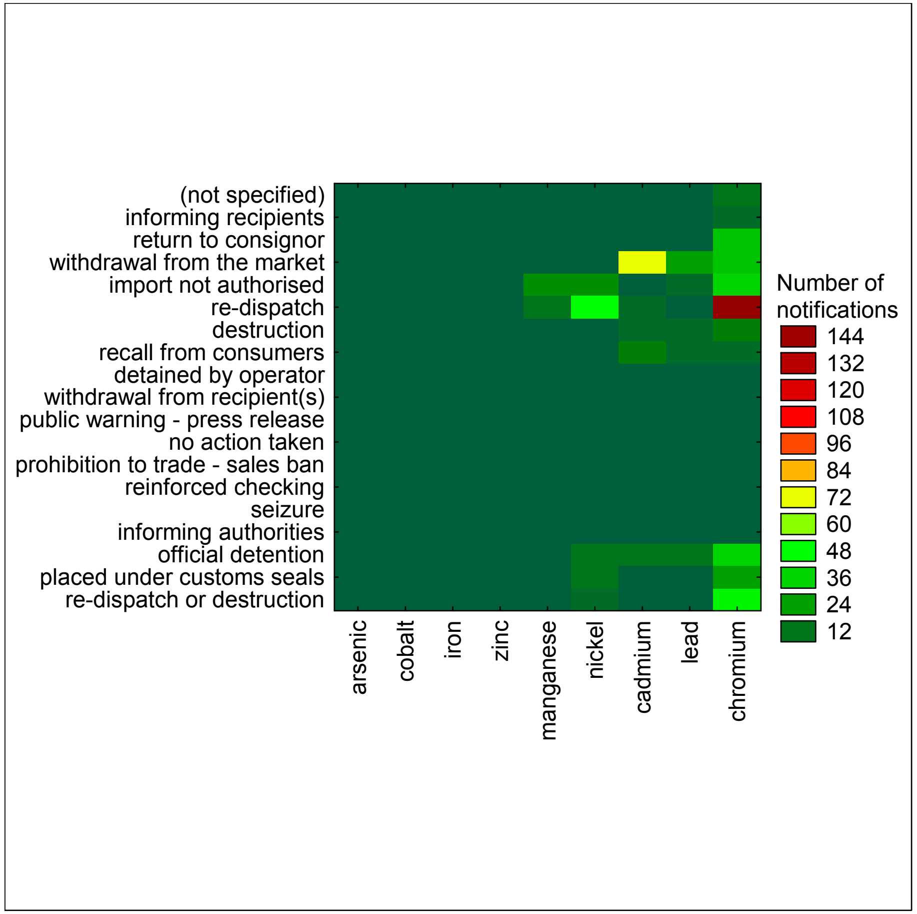

(**b**)

Figure S17: Similarities of RASFF notifications on heavy metals and action taken within food contact material: (**a**) joining; (**b**) two-way joining.

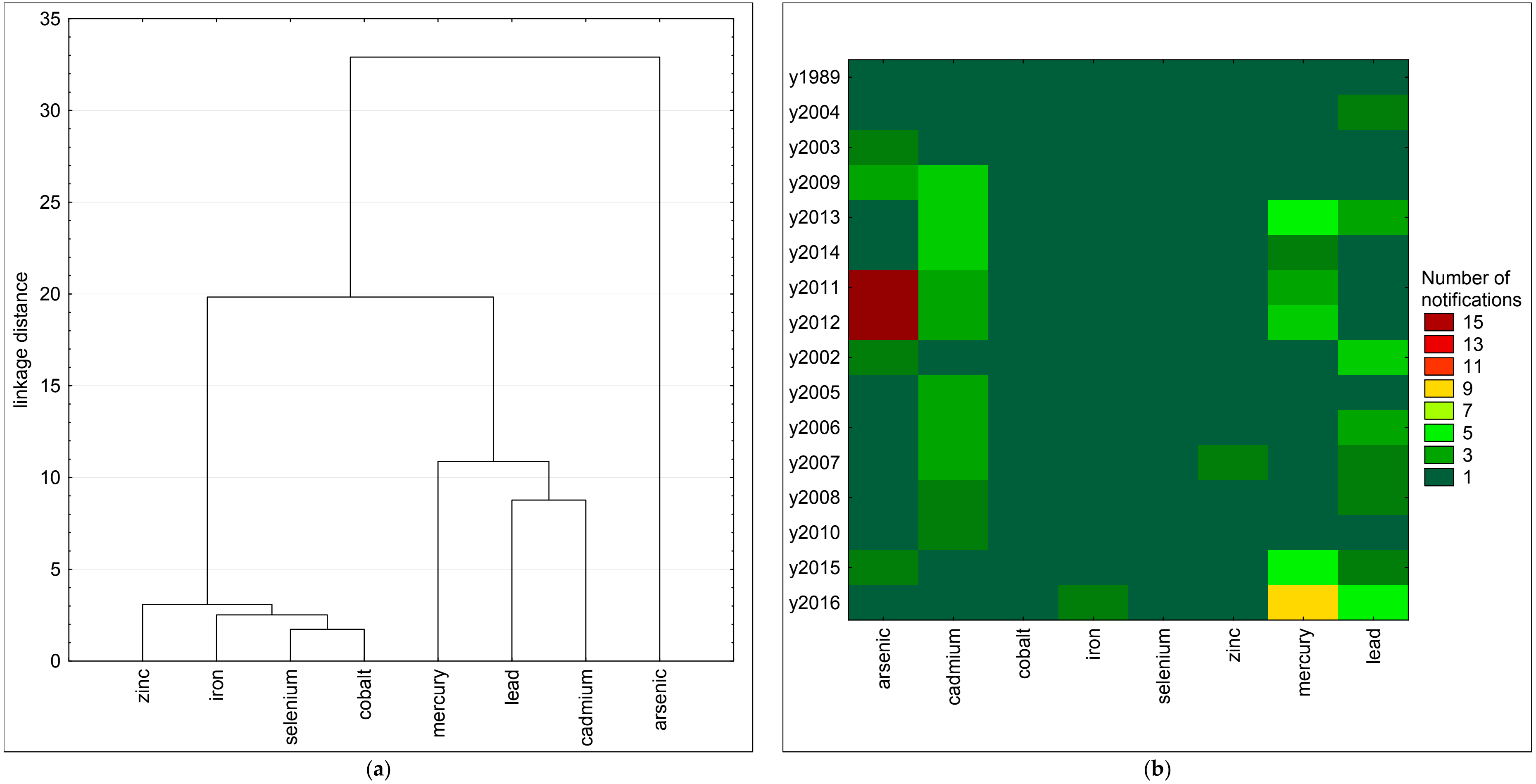

Figure S18: Similarities of RASFF notifications on heavy metals and year within food: (**a**) joining; (**b**) two-way joining.

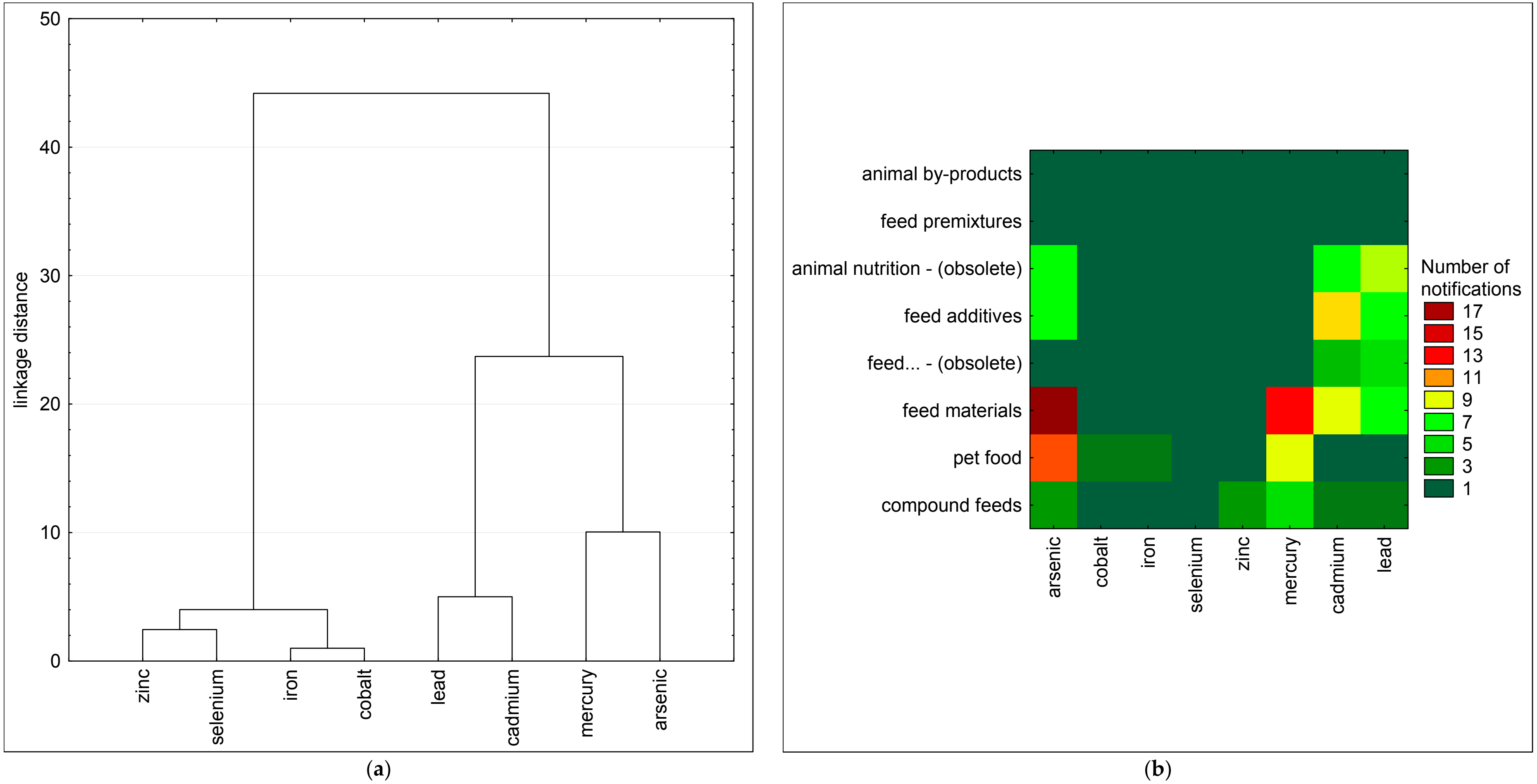

Figure S19: Similarities of RASFF notifications on heavy metals and product category within feed: (**a**) joining; (**b**) two-way joining.

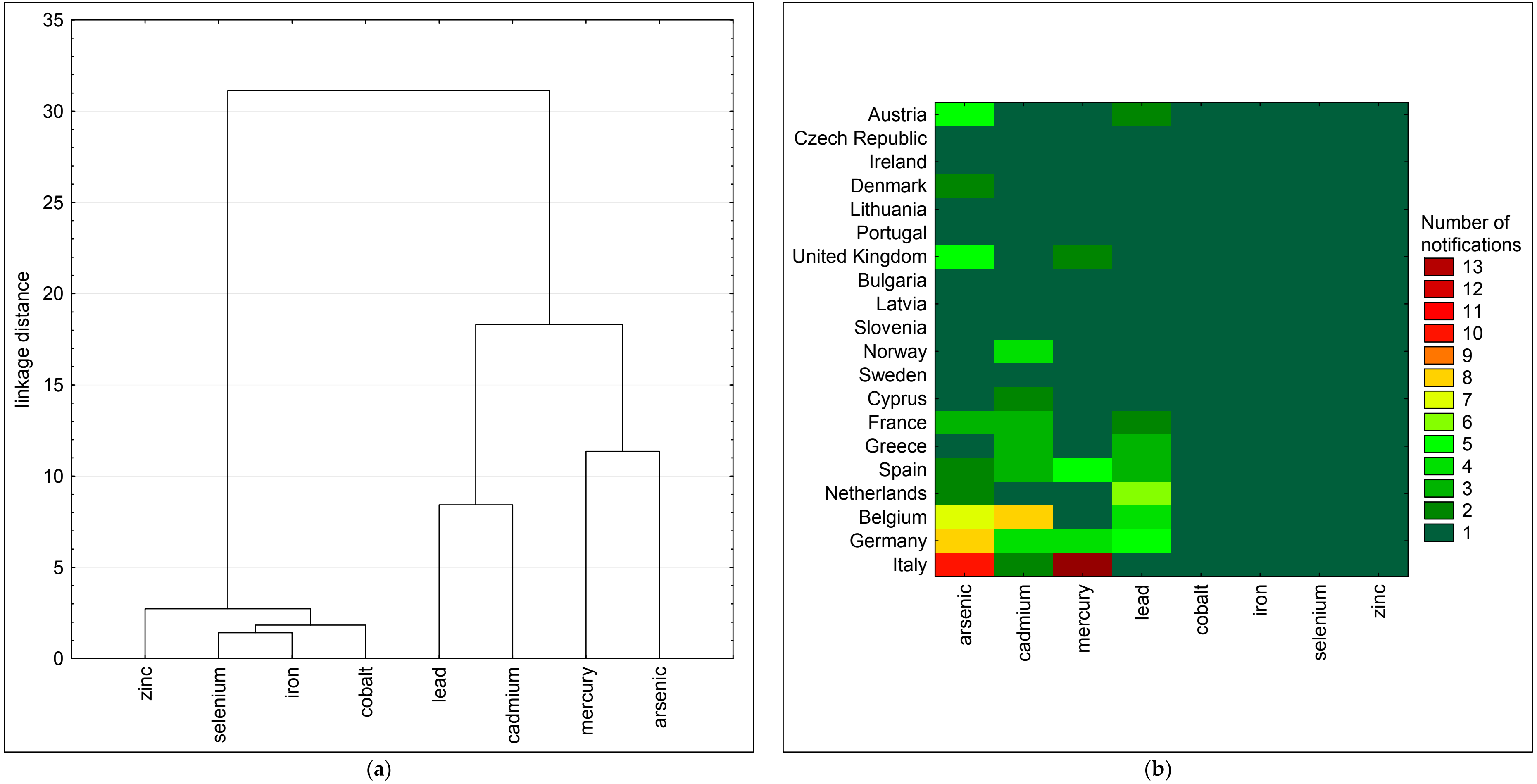

Figure S20: Similarities of RASFF notifications on heavy metals and notifying country within feed: (**a**) joining; (**b**) two-way joining.

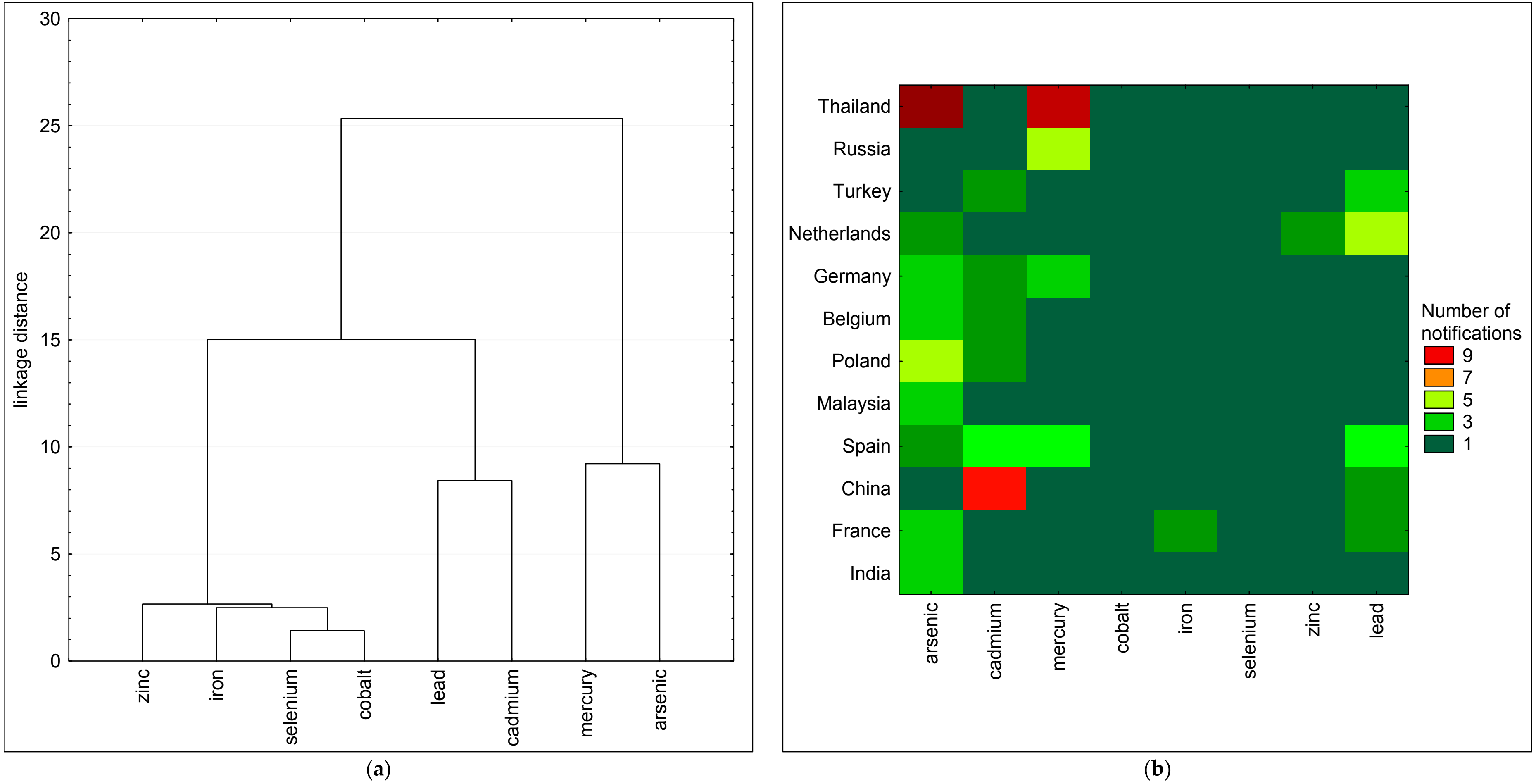

Figure S21: Similarities of RASFF notifications on heavy metals and origin country within feed: (**a**) joining; (**b**) two-way joining.

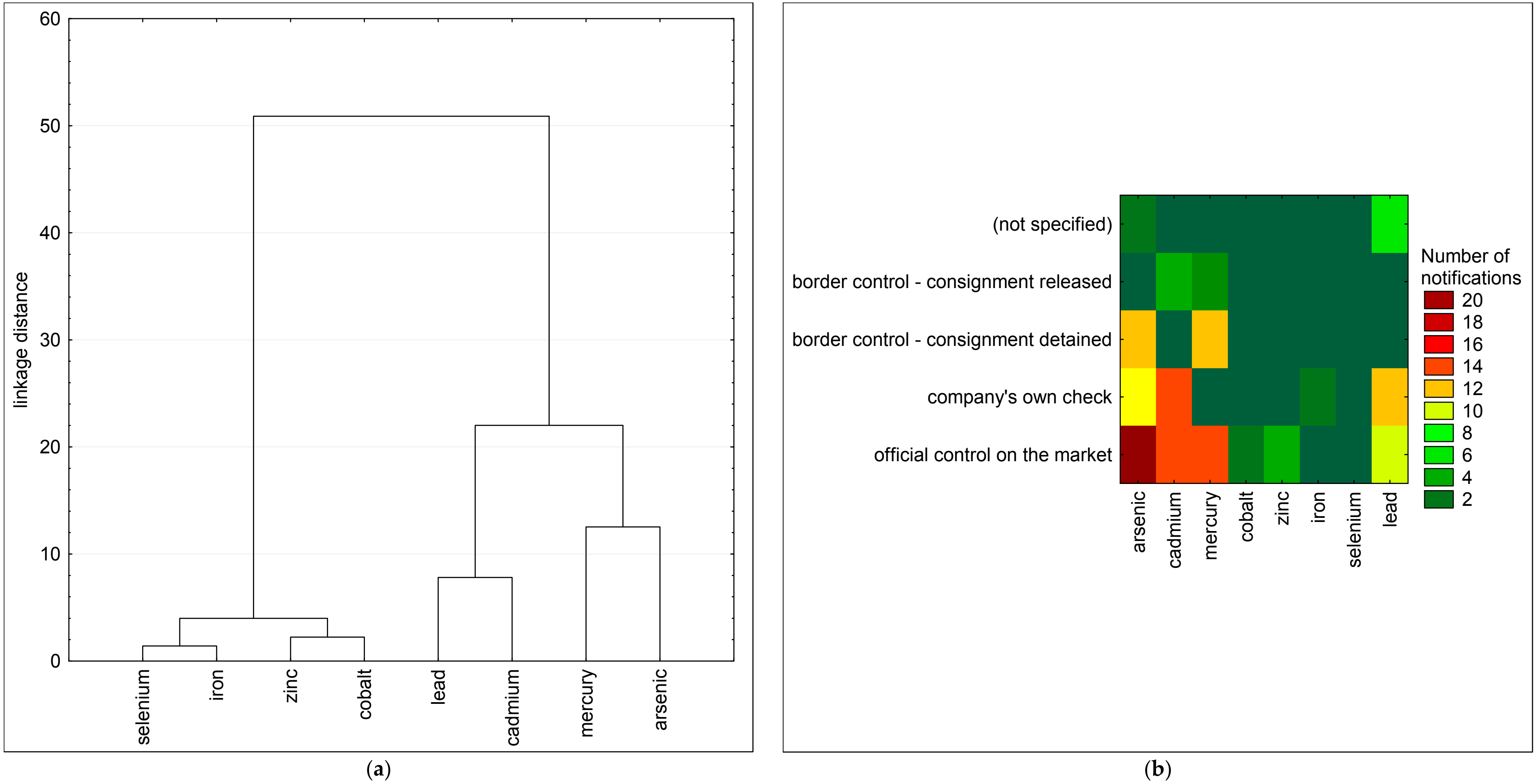

Figure S22: Similarities of RASFF notifications on heavy metals and notification basis within feed: (**a**) joining; (**b**) two-way joining.

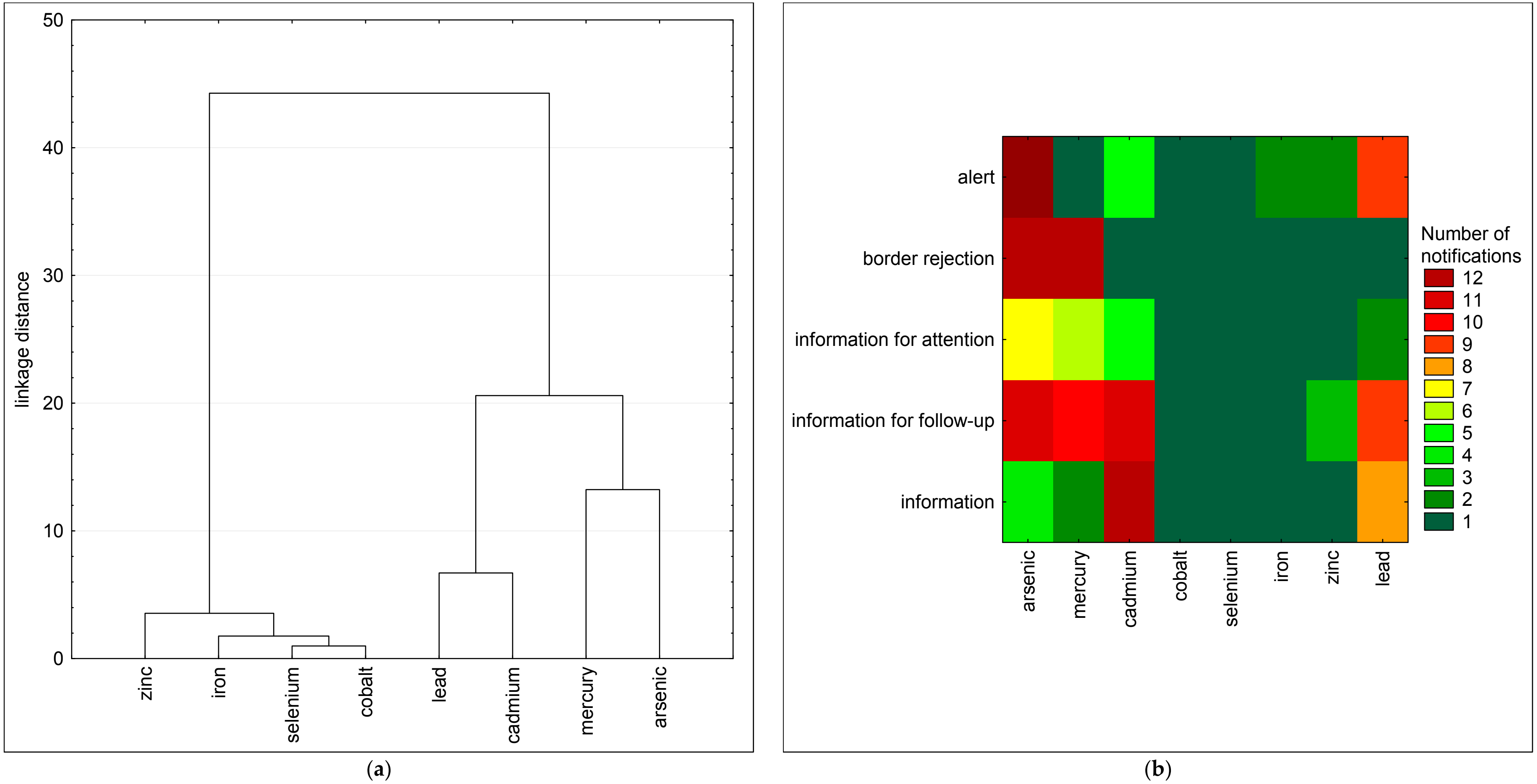

Figure S23: Similarities of RASFF notifications on heavy metals and notification type within feed: (**a**) joining; (**b**) two-way joining.

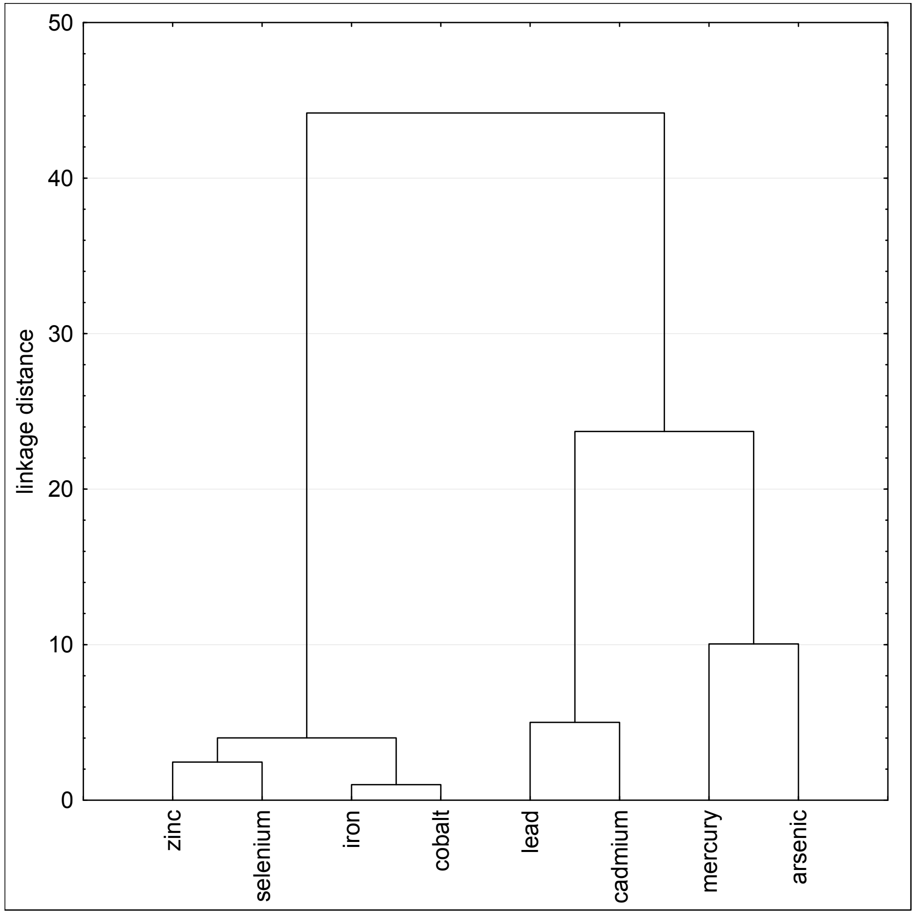

(**a**)

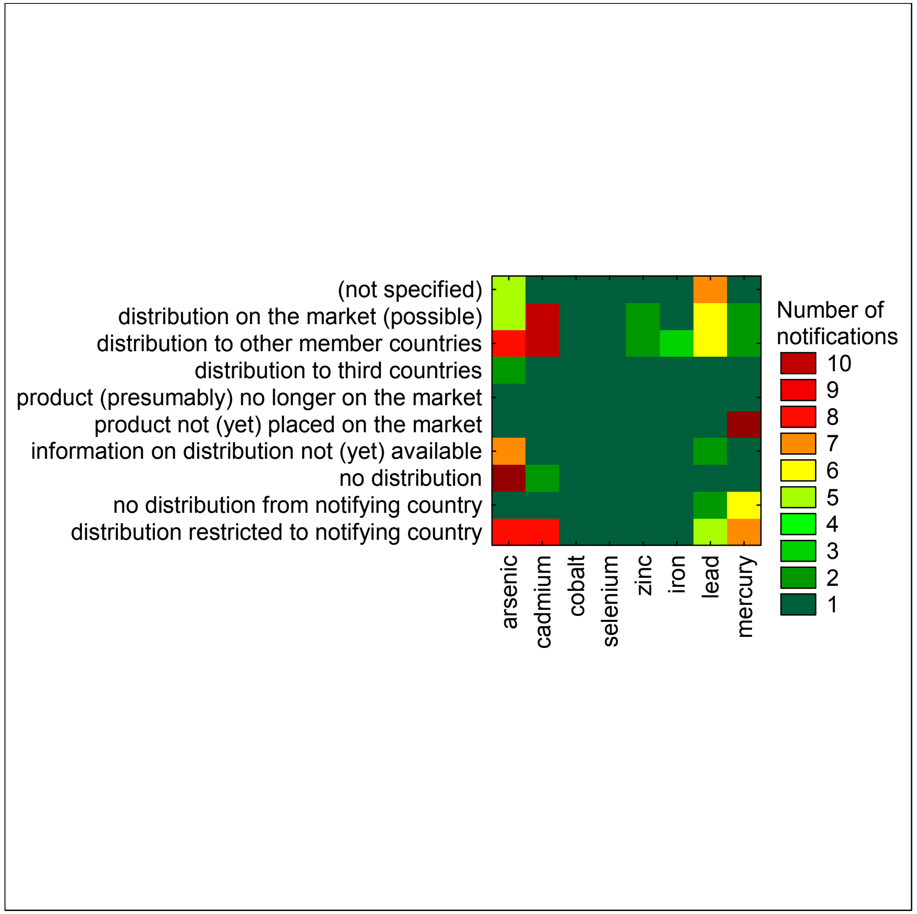

(**b**)

Figure S24: Similarities of RASFF notifications on heavy metals and distribution status within feed: (**a**) joining; (**b**) two-way joining.

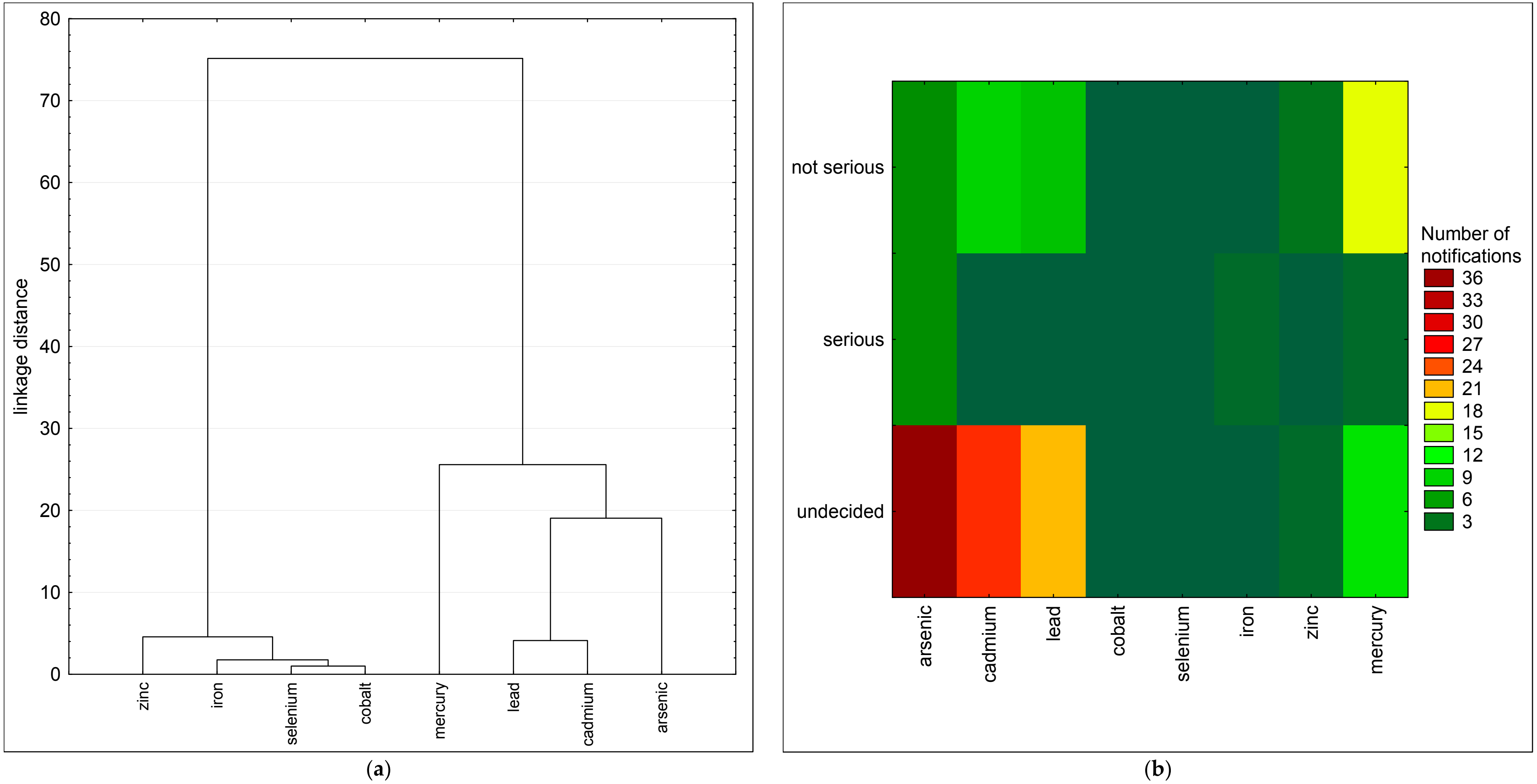

Figure S25: Similarities of RASFF notifications on heavy metals and risk decision within feed: (**a**) joining; (**b**) two-way joining.

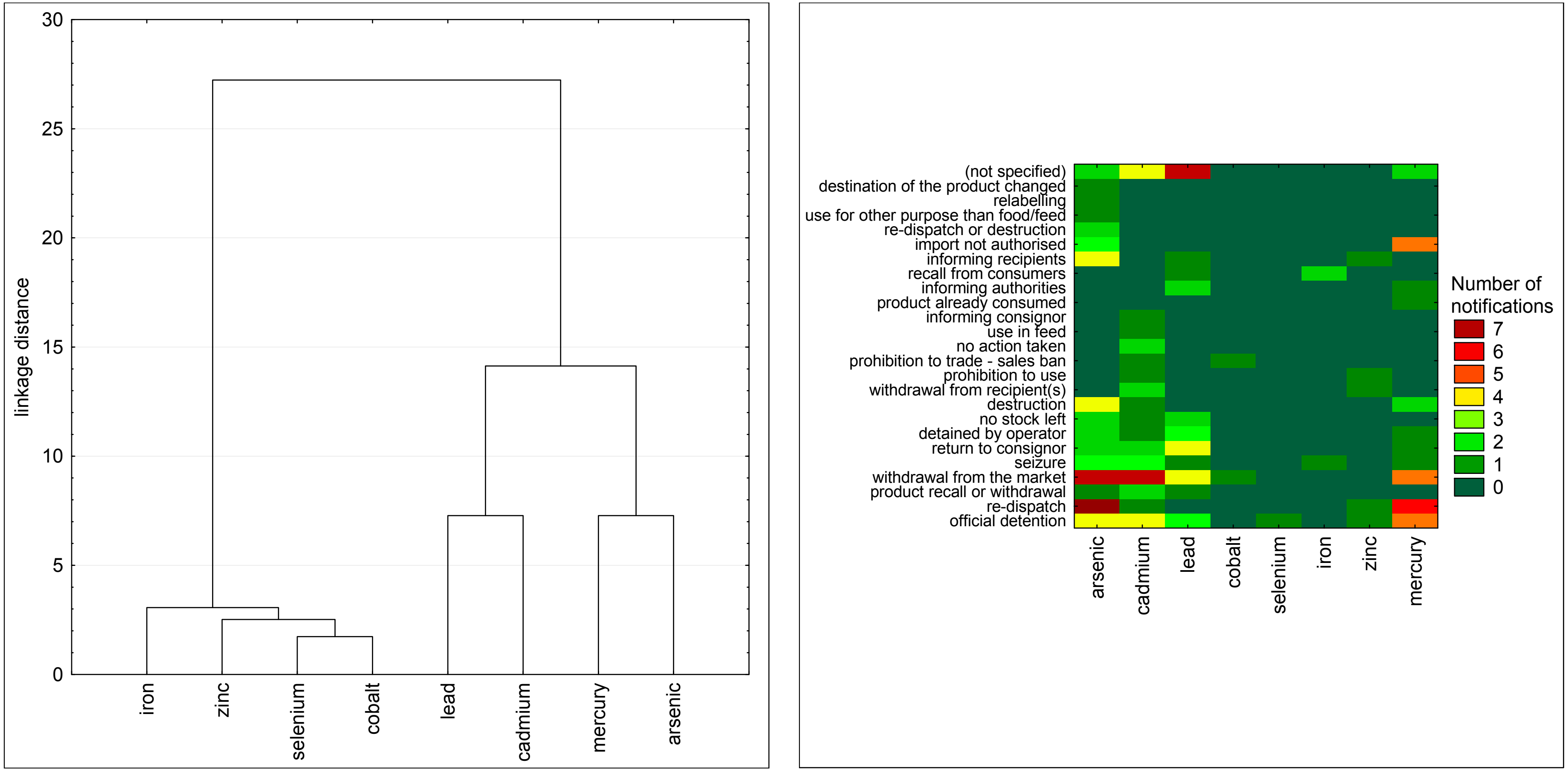

(**a**) (**b**)

Figure S26: Similarities of RASFF notifications on heavy metals and action taken within feed: (**a**) joining; (**b**) two-way joining.

© 2018 by the authors. Submitted for possible open access publication under the terms and conditions of the Creative Commons Attribution (CC BY) license (http://creativecommons.org/licenses/by/4.0/).
